# Biomechanics of Tooth-Supported Fixed Dental Prostheses: Material Systems, Connector Design, Retainer Design, and Abutment Stress Distribution—A Systematic Review of In Vitro and Finite Element Evidence

**DOI:** 10.3390/ma19132844

**Published:** 2026-07-03

**Authors:** Iuliana Babiuc, Andi Ciprian Drăguș, Viorel Ștefan Perieanu, Andrei Vorovenci, Andreea Angela Ștețiu, Mădălina Adriana Malița, Mihaela Romanița Gligor, Maria Antonia Ștețiu, Radu Cătălin Costea, Andrei Burlibașa, Mircea Popescu, Mihai Burlibașa

**Affiliations:** 1 Department of Dental Technology, Faculty of Midwifery and Nursing, Carol Davila University of Medicine and Pharmacy, 050474 Bucharest, Romania; iuliana.babiuc@umfcd.ro (I.B.); andi.dragus@drd.umfcd.ro (A.C.D.); viorel.perieanu@umfcd.ro (V.Ș.P.); madalina.malita@umfcd.ro (M.A.M.); radu-catalin.costea@umfcd.ro (R.C.C.); mihai.burlibasa@umfcd.ro (M.B.); 2 Doctoral School, Carol Davila University of Medicine and Pharmacy, 050474 Bucharest, Romania; 3 Faculty of Medicine, Lucian Blaga University of Sibiu, 550169 Sibiu, Romania; romanita.gligor@ulbsibiu.ro (M.R.G.); mariaantonia.stetiu@ulbsibiu.ro (M.A.Ș.); 4 Faculty of Medicine, Carol Davila University of Medicine and Pharmacy, 050474 Bucharest, Romania

**Keywords:** tooth-supported fixed dental prosthesis, zirconia, connector design, retainer design, resin-bonded fixed dental prosthesis, inlay-retained fixed dental prosthesis, fracture resistance, finite element analysis, abutment stress, stress distribution

## Abstract

**Highlights:**

**What are the main findings?**

- FDP behavior depends on both material and design.
- Connector size and shape affect fracture risk.
- Retainer design affects debonding and stress.
- Abutment support changes stress distribution.

**What are the implications of the main findings?**

- Material choice alone is not enough for FDP design.
- Connector design should be reported in detail.
- Conservative FDPs need careful retainer planning.
- FEA findings need validation before clinical use.

**Abstract:**

**Background**: Tooth-supported fixed dental prostheses (FDPs) remain relevant when implant therapy is limited, but their mechanical behavior depends on material selection, connector design, retainer design, prosthesis configuration, and abutment support. This systematic review assessed how these factors affect fracture behavior and stress transmission in tooth-supported FDPs. **Materials and Methods**: PubMed/MEDLINE, Scopus, Web of Science Core Collection, and Dentistry and Oral Sciences Source were searched for English-language studies published from 1 January 2016 to 15 May 2026. Eligible studies were in vitro mechanical, fatigue, fracture-resistance, or finite element analysis (FEA) studies of tooth-supported FDP designs. Clinical studies were screened during eligibility assessment, but no clinical study met the final inclusion criteria for primary synthesis. In vitro components were appraised with the Quality Assessment Tool for In Vitro Studies (QUIN), and FEA components were appraised with the Risk-of-bias Framework for Dental Finite Element Analysis (ROBFEAD). Findings were synthesized narratively by evidence type and biomechanical theme. **Results**: Twenty-nine studies were included: 11 in vitro-only studies, 14 FEA-only studies, and four combined experimental and computational studies. No eligible clinical study met the final inclusion criteria. Zirconia-based systems were the most frequent focus. Their behavior depended on connector dimensions, connector shape, framework design, span, retainer configuration, loading direction, abutment selection, periodontal support, and bone support. Larger connector dimensions or greater connector height often improved fracture resistance or reduced modeled stress in zirconia models, but connector area alone did not explain performance across all materials and designs. Conservative FDPs were sensitive to retainer geometry, adhesive-interface behavior, connector design, and abutment support. **Conclusions**: Current evidence is limited to laboratory and computational studies. Tooth-supported FDP biomechanics should be interpreted as a material, design, and support system, not as a material effect alone.

## 1. Introduction

Tooth-supported fixed dental prostheses (FDPs) remain a relevant treatment option for partially edentulous patients. While implant-supported restorations have influenced treatment planning, tooth-supported FDPs remain indicated when implants are contraindicated due to anatomical, medical, economic, or restorative limitations. A recent systematic review and meta-analysis reported 5-year survival estimates of 91.3% for metal-ceramic FDPs, 92.9% for veneered densely sintered zirconia FDPs, 87.9% for monolithic densely sintered zirconia FDPs, and 82.5% for lithium-disilicate reinforced glass-ceramic FDPs [1]. This evidence supports the ongoing clinical relevance of tooth-supported FDPs and indicates that material class alone does not fully account for clinical performance.

Metal-ceramic FDPs have traditionally served as the reference standard due to their established clinical record and mechanical reliability [2,3,4]. Biological considerations related to base-metal dental alloys also remain relevant when discussing material selection in fixed prosthodontics [5]. This background supports continued interest in zirconia and other ceramic systems for tooth-supported FDPs [6,7]. Zirconia-based frameworks were introduced because yttria-stabilized tetragonal zirconia polycrystal provides greater strength and fracture toughness than many glass-based ceramics [2,6,7,8]. However, early zirconia FDPs often required veneering ceramics to improve esthetics, and chipping of these veneers appeared as a frequent technical complication. One review from 2018 reviewed 430 zirconia FDPs in 368 patients and reported a mean survival rate of 89.43% ± 10.01%, with chipping of the veneering ceramic observed in 16.97% of cases [7]. It was subsequently reported that minor ceramic chipping occurred after five years in 17.3% of veneered zirconia FDPs and 4.4% of monolithic zirconia FDPs [1,9]. These results show that framework material, veneering strategy, and prosthesis design must be evaluated collectively.

Lithium disilicate is widely used in esthetic tooth-supported restorations and serves as an important comparator for ceramic FDP design. However, the clinical evidence differs between single crowns, partial FDPs, and complete-coverage prostheses. Pieger et al. reported more favorable clinical evidence for lithium disilicate single crowns than for lithium disilicate partial fixed dental prostheses [10]. A later review of complete-coverage tooth-retained lithium disilicate prostheses also indicated that survival estimates vary by prosthesis type, follow-up duration, tooth region, and study design [11]. These findings highlight the importance of considering span, tooth region, prosthesis design, and loading conditions when selecting a material.

Conservative tooth-supported FDP designs bring additional biomechanical complexity. Conventional full-coverage FDPs often demand considerable removal of sound coronal tooth structure. Studies reported preparation values ranging from 67.5% to 75.6% for full crowns in conventional FDPs [12,13]. Alternative designs, such as resin-bonded FDPs, inlay-retained FDPs, wing-retained FDPs, and cantilever FDPs, were developed to minimize tooth preparation, but these approaches carry risks associated with adhesive retention, connector fracture, retainer geometry, and abutment loading. A systematic review estimated 3-year and 5-year survival rates of 92.6% and 87.9%, respectively, with debonding, veneer fracture, dentine hypersensitivity, and secondary caries identified as primary complications [12]. For all-ceramic resin-bonded FDPs, an estimated 5-year survival rate of 91.2% was reported, with debonding and framework fracture as the most frequent technical complications [14]. Because conservative FDPs rely on adhesive retention, bonding protocol and dentin-interface stability remain relevant background factors when interpreting debonding risk in resin-bonded and inlay-retained designs [15].

Retainer configuration also influences stress transfer within FDPs. Mourshed et al. reviewed anterior cantilever resin-bonded FDPs and identified single-retainer designs as minimally invasive options that avoid the differential mobility issues associated with splinting two abutment teeth [3]. Furthermore, cantilevered all-ceramic resin-bonded FDPs demonstrated higher survival and lower rates of debonding and fracture compared to two-retainer all-ceramic resin-bonded FDPs [14]. Mendes et al. estimated 5-year survival rates of 91.9% for cantilever anterior resin-bonded FDPs and 85.2% for two-wing designs, although this difference was not statistically significant [16]. These outcomes do not identify a universally preferable design but point out the importance of evaluating retainer design as a biomechanical variable.

Connector design represents a central factor in the biomechanics of FDPs. Both clinical and laboratory studies frequently identify the connector region as a site of stress concentration and fracture. Raigrodski et al. summarized clinical zirconia FDP studies in which connector dimensions varied, reporting connector areas of at least 9 mm^2^, 6 to 9 mm^2^, and 15 to 16 mm^2^ across different designs [6]. In a finite element analysis of anterior ceramic resin-bonded FDPs, Mohd Osman et al. modeled lithium disilicate and zirconia prostheses with varying connector height, width, and shape parameters under oblique loading of 100 N, 150 N, and 200 N. The study found that material type, connector dimensions, connector shape, and load magnitude all influenced both the maximum equivalent stress and predicted connector integrity [17]. While these outcomes are computational instead of direct clinical evidence, they demonstrate the relevance of connector geometry to failure mode.

The objective of this paper is to synthesize evidence regarding the biomechanical behavior of tooth-supported FDPs, with particular emphasis on material combinations, connector design, retainer design, and stress distribution to abutment teeth and supporting tissues. While clinical reviews provide context for current material functionality and complication patterns, they do not isolate the mechanical effects of connector geometry, retainer configuration, framework thickness, or abutment support. Hence, this review prioritizes in vitro mechanical studies and finite element analyses. Findings from laboratory and computational studies are interpreted as biomechanical evidence.

## 2. Materials and Methods

### 2.1. Review Design

Systematic literature searching and stratified narrative synthesis were used. The protocol was prepared before final synthesis but was not prospectively registered. Reporting followed the Preferred Reporting Items for Systematic Reviews and Meta-Analyses (PRISMA) 2020 guidance [18]. Clinical studies were screened during eligibility assessment only when they evaluated an eligible tooth-supported fixed dental prosthesis design and a relevant biomechanical design variable. The completed PRISMA checklist is provided as Supplementary Material File S1. During manuscript revision, the protocol and supporting review documents were deposited in the Open Science Framework as a non-prospective protocol deposit: https://osf.io/cu94b/overview?view_only=1ef11857d5e04a6ebdc78c2b26756d50 (accessed on 16 June 2026).

### 2.2. PICO Framework

The review addressed the following question: In tooth-supported fixed dental prostheses, how do material selection, connector design, retainer design, and prosthesis configuration affect mechanical performance and stress transmission to abutment teeth and supporting tissues?

The review question and eligibility process were structured using a PICO framework summarized in Table 1.

### 2.3. Eligibility Criteria

Eligibility criteria are outlined in Table 2. The search was restricted to studies published within the previous 10 years, from 1 January 2016 to 15 May 2026. The included models consisted of extracted-tooth, artificial-tooth, and computational models of tooth-supported fixed dental prostheses. Eligible prosthesis designs included conventional full-coverage tooth-supported fixed dental prostheses, resin-bonded fixed dental prostheses, inlay-retained fixed dental prostheses, wing-retained fixed dental prostheses, cantilever tooth-supported fixed dental prostheses, and anterior or posterior tooth-supported bridge designs.

Clinical studies were eligible during initial screening only if they evaluated an eligible tooth-supported fixed dental prosthesis design and a relevant biomechanical design variable. Implant-supported prostheses, tooth-implant-supported prostheses, removable prostheses, overdenture designs, implant bars, and material coupon studies without a fixed dental prosthesis design were excluded. Systematic and narrative reviews were not considered primary evidence but were used for background information and source checking when relevant.

### 2.4. Information Sources

Electronic searches were conducted in PubMed/MEDLINE, Scopus, Web of Science Core Collection, and Dentistry and Oral Sciences Source. Supplementary sources included reference lists from included studies, reference lists from relevant reviews, forward citation tracking of key studies, targeted Google Scholar checking, and Elicit Pro (Elicit, https://elicit.com; accessed on 15 May 2026). Elicit Pro (Elicit Research, PBC, Covina, CA, USA) was used only for supplementary record discovery and cross-checking after the main database searches. Candidate records identified through supplementary routes were screened against the same eligibility criteria as database records, checked against the deduplicated reference set, and assessed by full text when available. No Elicit-derived record or data item was used as primary evidence unless it was verified against the full text, audit table, or structured extraction files. Unverified records were excluded from the synthesis or marked as requiring full-text verification.

### 2.5. Search Strategy

The search strategy combined terms related to prosthesis type, tooth support, restorative material, and biomechanical outcome. Search strings were adapted to the syntax of each database. The database-specific search syntax is presented in Table 3.

A supplementary conservative-design search was carried out to increase the retrieval of records for resin-bonded, inlay-retained, wing-retained, and cantilever fixed dental prostheses. The supplementary search syntax is presented in Table 4.

### 2.6. Study Selection

All records were exported from the searched databases, and duplicate records were removed before screening. Titles and abstracts were screened against the predefined eligibility criteria. Records unrelated to tooth-supported fixed dental prostheses, implant-supported only, removable prosthodontic designs, case reports, narrative reviews, editorials, letters, or isolated material tests without a fixed dental prosthesis design were excluded.

Full texts were retrieved for records that appeared potentially eligible or could not be excluded based on title and abstract information alone. Full-text articles were assessed against the inclusion and exclusion criteria. Reasons for exclusion at the full-text stage were recorded and grouped for PRISMA reporting.

Screening was performed according to the predefined protocol. Four independent reviewers screened titles/abstracts against the eligibility criteria. Full texts were retrieved for potentially eligible records and independently assessed by four reviewers. Disagreements were resolved by discussion; a fifth reviewer adjudicated when required.

### 2.7. Data Extraction

Data were extracted using structured tables. For all included studies, the following general fields were recorded: author, year, country, study design, prosthesis type, tooth region, missing tooth or span, abutment configuration, material or materials, comparator, sample size, manufacturing method, cementation or bonding protocol, testing protocol, primary outcomes, main results, failure modes, limitations, and synthesis notes.

For in vitro studies, additional extracted fields included specimen type, sample size per group, prosthesis design, material, manufacturing method, connector dimensions, framework thickness, retainer design, aging protocol, thermocycling protocol, mechanical loading protocol, fracture load, fatigue outcome, failure mode, fracture origin, and statistical findings.

For finite element analysis studies, additional extracted fields included model source, anatomical region, abutment configuration, prosthesis design, material properties, connector dimensions, periodontal ligament modeling, bone modeling, mesh details, boundary conditions, loading magnitude, loading direction, outcome measures, peak stress location, validation method, main assumptions, and limitations.

Missing data were recorded as not reported. Extracted findings were maintained as study-specific. Numerical values, sample sizes, fracture loads, stress values, failure modes, and statistical findings were not inferred when not explicitly reported.

Data extraction was performed by four reviewers, I.B., A.C.D., A.V., and A.A.Ș., using structured extraction tables. Extracted data were checked by V.Ș.P. and M.B. Disagreements, uncertain data fields, and inconsistent extraction items were resolved by discussion. Missing data were recorded as not reported. Numerical values, sample sizes, fracture loads, stress values, failure modes, and statistical findings were not inferred when not explicitly reported.

### 2.8. Methodological Appraisal

Methodological appraisal was performed by the evidence component. For studies with both experimental and computational components, the in vitro and finite element analysis components were appraised separately. In vitro studies and experimental components were appraised using the Quality Assessment Tool for In Vitro Studies (QUIN) [19]. Finite element analysis components were appraised using the Risk-of-bias Framework for Dental Finite Element Analysis (ROBFEAD) [20]. Photoelastic components used only for qualitative validation of finite element stress patterns were recorded as validation evidence and were not converted into standalone QUIN judgments. Methodological appraisal was performed independently by three reviewers, M.A.M., M.R.G., and M.A.Ș. Disagreements were resolved by discussion with M.B. and R.C.C. Appraisal results were reported by evidence type and were not combined into a single numerical score.

Judgments were reported as low concern, some concerns, or high concern according to predefined domain-level appraisal decisions. For finite element analysis studies, the appraisal also considered whether each study reported the model source, geometry creation method, material properties, mesh information, convergence testing, boundary conditions, loading conditions, validation, and sensitivity analysis.

### 2.9. Data Synthesis

A stratified narrative synthesis was planned. In vitro mechanical studies and finite element analysis studies were synthesized separately. Quantitative pooling was not planned because heterogeneity was expected in prosthesis designs, materials, connector dimensions, abutment configurations, aging protocols, loading conditions, outcome measures, and finite element modeling assumptions.

The synthesis was organized around five themes: in vitro mechanical performance of tooth-supported fixed dental prostheses, conservative retainer concepts, connector and framework biomechanics, abutment stress distribution, and research gaps with future experimental directions. Laboratory and computational findings were not treated as clinical evidence.

### 2.10. Use of Artificial Intelligence and Generative Artificial Intelligence Tools

As described above, Elicit Pro (Elicit Research, PBC, Covina, CA, USA) was used for supplementary record discovery and cross-checking. ChatGPT (GPT-5.5 Thinking, OpenAI, San Francisco, CA, USA) was used for drafting support, text restructuring, language refinement, and protocol consistency checks. Grammarly (v.1.2.259.1886, Superhuman Platform Inc., San Francisco, CA, USA) was used for grammar, spelling, and clarity checks. These tools did not replace database searching, duplicate removal, eligibility decisions, data extraction, methodological appraisal, synthesis, or interpretation. All records, extracted data, appraisal judgments, and manuscript claims were manually verified against full texts, audit files, extraction tables, or cited background sources before inclusion. The authors reviewed and edited all tool-assisted outputs and take full responsibility for the content of the manuscript.

## 3. Results

### 3.1. Search Results

**Figure 1:**
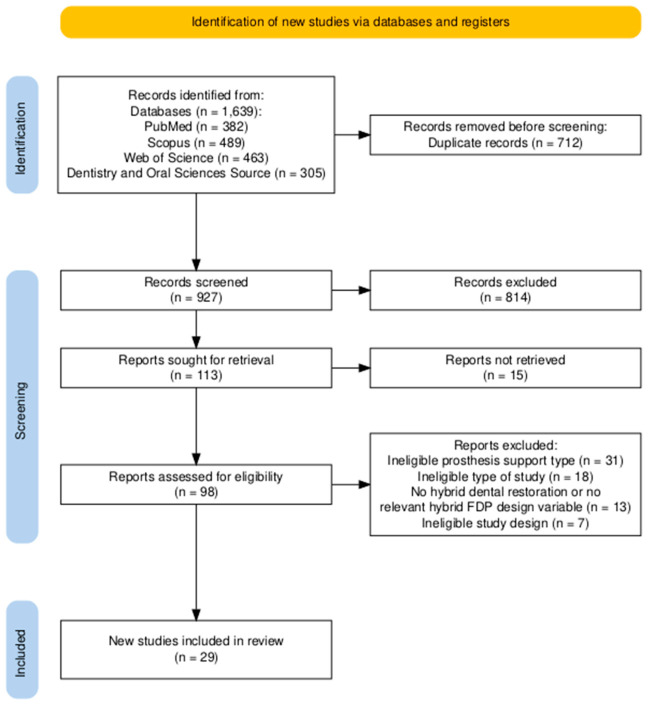
PRISMA flow diagram [21].

The electronic database searches retrieved 1639 records: 382 from PubMed/MEDLINE, 489 from Scopus, 463 from Web of Science Core Collection, and 305 from Dentistry and Oral Sciences Source, EBSCO. After removing 712 duplicates, 927 records were screened by title and abstract. Of these, 814 records were excluded, and 113 reports were sought for retrieval. Fifteen reports could not be retrieved, leaving 98 full-text reports for eligibility assessment. After full-text assessment, 69 reports were excluded: 31 because of ineligible prosthesis support type, 18 because of ineligible article type, 13 because they did not evaluate an eligible tooth-supported restoration or a relevant biomechanical design variable, and 7 because of ineligible study design. Twenty-nine studies were included in the review (Figure 1).

The final set comprised 11 in vitro-only studies, 14 finite element analysis-only studies, and 4 studies that combined experimental and finite element analysis components. No eligible clinical study met the final inclusion criteria, so clinical outcome data were not synthesized as primary evidence. The final primary evidence base was therefore limited to in vitro studies of mechanical testing, fatigue, fracture resistance, and finite element analysis.

### 3.2. Methodological Appraisal Results

**Figure 2:**
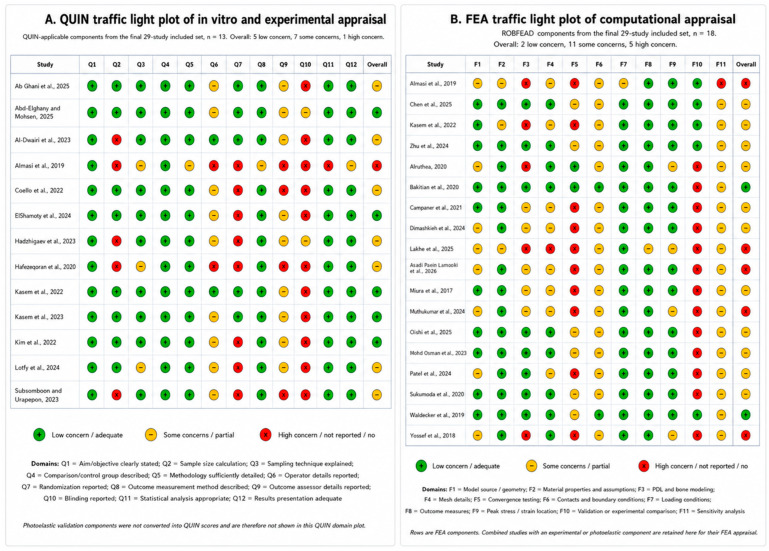
Study-level traffic light plots for methodological appraisal. Panel (**A**) shows QUIN judgments for in vitro and experimental components [22,23,24,25,26,27,28,29,30,31,32,33,34]. Panel (**B**) shows ROBFEAD judgments for finite element analysis components [13,17,24,34,35,36,37,38,39,40,41,42,43,44,45,46,47,48].

Methodological appraisal was completed for the final 29 included studies (Figure 2). Because the final evidence base contained only in vitro mechanical studies, experimental validation components, and finite element analysis components, clinical risk-of-bias tools were not applied. The appraisal results were reported by evidence type. QUIN was applied to 13 in vitro or experimental components. Two photoelastic validation components were recorded as validation evidence and were not converted into QUIN judgments. ROBFEAD was applied to 18 finite element analysis components. The appraisal results were not merged into a single numerical score.

For the QUIN-applicable components, 5 of 13 were judged as low concern, 7 as some concerns, and 1 as high concern. Low concern was assigned to studies with clear aims, defined comparison groups, sufficiently detailed methods, appropriate outcome measurement, and adequate statistical reporting [22,23,24,25,26]. Some concerns were assigned when studies met the main design and reporting criteria but had partial or absent reporting in domains such as operator details, randomization, blinding, outcome assessor details, or sample-size justification [27,28,29,30,31,32,33]. One in vitro component was judged as high concern because it used one specimen per connector design, did not report sample-size calculation or randomization, did not provide inferential statistics, and used a simplified testing configuration [34].

The two photoelastic validation components were not scored as standalone QUIN. They were retained as supporting validation evidence for computational stress patterns, not as independent laboratory outcome studies [13,35]. These components were therefore considered useful for interpreting modeled stress distribution, but they were not treated as clinical validation.

For the finite element analysis components, 2 of 18 were judged as low concern, 11 as some concerns, and 5 as high concern. Low concern was assigned to studies with more complete reporting of model source or geometry, material assumptions, periodontal ligament and bone modeling, mesh details, loading conditions, outcome measures, and stress-location reporting [36,37]. Some concerns were assigned to most FEA components because several domains were reported only partially, especially convergence testing, contact definitions, boundary conditions, validation, or sensitivity analysis [13,17,24,38,39,40,41,42,43,44]. High concern was assigned when the computational model had major reporting or modeling limitations, including unclear model source, incomplete periodontal or bone modeling, absent convergence testing, limited validation, or results that required cautious interpretation.

The most frequent limitations in the in vitro evidence were the absence or partial reporting of blinding, outcome assessor details, randomization, and sample size calculation. The most frequent limitations in the FEA evidence were the absence of validation, limited reporting of convergence, partial sensitivity analysis, and simplified contact or boundary assumptions. These limitations affected the level of confidence placed in individual findings, but they did not lead to exclusion from the final synthesis when the study otherwise addressed an eligible tooth-supported FDP question.

The appraisal decisions were used to guide synthesis weighting. Low-concern studies were treated as the most reliable preclinical evidence within their evidence type. Studies with some concerns were retained for the main synthesis, but their findings were interpreted with methodological caution. High-concern studies were retained only as limited or supporting evidence and were not used as stand-alone support for design recommendations. Overall, the appraisal profile supports a cautious interpretation of the body of evidence. The included studies provide laboratory and computational evidence on material selection, connector design, retainer configuration, and abutment stress distribution, but they do not provide clinical proof of survival, complication reduction, or long-term performance.

### 3.3. Evidence Profile

The final evidence base included 29 studies. No eligible clinical study met the final inclusion criteria; therefore, the synthesis was limited to laboratory and finite element evidence. The retained studies covered conventional three-unit fixed dental prostheses, cantilever resin-bonded fixed dental prostheses, inlay-retained designs, endocrown-related designs, and long-span anterior and posterior configurations. Some studies combined experimental and computational methods, which allowed direct comparison between observed fracture behavior or photoelastic patterns and modeled stress maps.

Across the included studies, the main variables were material system, connector dimensions and geometry, retainer design, span configuration, and abutment or periodontal support. Most studies evaluated zirconia-based restorations, either as the primary material or as the main comparator, within tooth-supported FDP models rather than implant-supported systems (Table 5).

### 3.4. In Vitro Mechanical Performance

The in vitro studies mainly reported fracture load, fatigue survival, retention, or failure mode. Across these studies, larger connector dimensions were often associated with higher failure loads, although the size of the effect varied by prosthesis design, loading condition, and test method [23,26,27,31]. When the failure location was reported, fractures often initiated at or near the connector region rather than in the retainer body or pontic surface.

Connector geometry also affected performance. Rounded or less-acute embrasure forms were associated with lower stress concentrations or higher load capacities in several zirconia models, although not all pairwise comparisons were statistically significant [31,32,34]. Studies that held the connector area constant still reported differences related to height, width, or aspect ratio, which means the connector area alone did not capture the full mechanical effect of design.

Long-span and cantilevered configurations remained mechanically demanding in laboratory models. In long-span anterior zirconia FDPs, fatigue failures still occurred under cyclic loading despite connector modifications, and the reported effect of connector size was smaller than that of the overall configuration in some test settings [29]. This pattern supports a cautious reading of connector data from simplified fracture tests, because span and support conditions changed the mechanical response of the same material system.

### 3.5. Conservative Retainer Findings

The conservative designs did not produce a single pattern across all models. In cantilever resin-bonded fixed dental prostheses and inlay-retained FDPs, retainer configuration influenced debonding, fatigue survival, or fracture behavior, while material comparisons were not uniformly significant across all design variants [24,25,28]. For cantilever inlay-retained zirconia FDPs, differences in fatigue behavior and failure mode were reported, but final fracture-load differences were not consistent across all preparation and material combinations [28].

Retainer form also affected measured retention. In one mandibular zirconia bridge model, full-coverage retainers produced higher tensile bond strength than the other tested retainer designs, but this result reflects pull-off retention rather than occlusal fracture resistance [22]. In posterior monolithic zirconia models, changes in distal abutment preparation also altered fracture behavior, although the available data did not support a simple claim that one preparation design was always better under all conditions [30].

Finite element studies on conservative designs reached a similar conclusion. In anterior cantilever zirconia resin-bonded FDP models, canine abutments and larger connectors were associated with lower framework strain, periodontal ligament strain, cement shear stress, and modeled debonding-risk area than central incisor abutments or smaller connectors [43]. In posterior resin-bonded and endocrown-related models, retainer design changed stress distribution in the restoration, abutment, and surrounding supporting structures, which means the mechanical effect of conservative treatment concepts depended on both the prosthesis design and the local support condition.

### 3.6. Connector and Framework Biomechanics

Connector cross-sectional area was among the most frequently repeated design variables across the full evidence set. Larger connector areas usually lowered peak connector stress in finite element models or increased fracture resistance in experimental models, but the threshold at which this occurred varied by material, span length, and prosthesis design [27,34,35,45]. The combined studies were consistent with this pattern because areas of high modeled stress corresponded to observed fracture origins or stress concentrations in the associated experimental or photoelastic component.

Material effects were reported across both in vitro and computational studies, but the direction of the effect depended on the outcome being measured. In some models, zirconia produced lower simulated stresses or higher fracture resistance than comparator materials, but these differences changed with connector geometry, framework configuration, and loading setup [25,32,33,35]. The included evidence, therefore, supports a context-specific material effect rather than a single material ranking that applies across all tooth-supported FDP designs.

Framework design also altered stress distribution. Finite element models that compared monolithic, semi-monolithic, veneered, or modified framework concepts reported changes in the location and magnitude of framework, veneer, and interface stresses when support geometry or veneer support pattern was altered [36,40,41]. Span length had a similar effect. Longer-span posterior and cantilevered models produced higher reported stresses than shorter or less-extended configurations, indicating that connector and framework findings need to be read together with the span configuration rather than as isolated variables.

### 3.7. Abutment Stress Distribution

Finite element analysis studies concentrated on how abutment support, periodontal conditions, and local geometry changed load transfer to the tooth and supporting tissues. Reduced alveolar bone support increased framework, cement, or periodontal stress in anterior resin-bonded models, which indicates that support reduction changed both prosthesis behavior and tissue-level loading in the tested configurations [42,44]. In the same general group of anterior zirconia models, canine abutments produced lower risk-related stress or strain measures than central incisor abutments under otherwise similar loading assumptions [43].

Abutment geometry also affected stress concentration. In models with tilted distal abutments, greater inclination increased local stress concentration, and the photoelastic component followed the same qualitative direction as the finite element output [13]. In endodontically treated abutment models restored beneath zirconia FDPs, the substrate or core condition also altered stress transmission, indicating that the abutment itself remained an active design factor rather than a passive support element [39].

These findings were replicated across multiple finite element studies, but they remained model-based. The included evidence supports a relationship between support condition and stress distribution in tooth-supported FDP models, yet those results should still be read as computational evidence rather than direct clinical outcome data.

### 3.8. Evidence Limits

The retained evidence was indirect and method-sensitive. One validation-focused study reported that simulated abutment material and support condition changed estimated fracture resistance substantially, which indicates that laboratory setup itself was a source of heterogeneity across in vitro findings [37]. This matters for interpretation because differences between studies could arise from support simulation as well as from the prosthesis design being tested.

The combined experimental and computational studies improved internal consistency within the evidence base because fracture origin, photoelastic stress patterns, and finite element stress maps generally pointed to similar high-stress regions, especially at connectors or at mechanically vulnerable support areas [13,34,35]. Even so, no clinical study met the final inclusion criteria. The results, therefore, define biomechanical trends in laboratory and computational settings, not clinical superiority among FDP materials or designs. Accordingly, the included evidence positions this review as a preclinical biomechanical synthesis based on laboratory and computational findings, rather than as a clinical comparative review of material or design superiority.

### 3.9. Synthesis of Biomechanical Findings

Across the included evidence, the most consistent biomechanical finding was the effect of connector design. Larger connector dimensions, increased connector height, rounded or less acute embrasure transitions, and favorable height-to-width relationships often improved fracture resistance or reduced modeled stress in the tested configurations [22,25,26,30,33,34,44]. However, connector area alone did not explain performance across all designs, because span length, connector shape, framework thickness, material stiffness, loading direction, and support conditions modified the effect [25,28,32,44].

A second consistent pattern was the role of span and support. Longer spans, cantilever configurations, reduced alveolar bone support, inclined abutments, and altered abutment substrate conditions increased mechanical demand or changed stress distribution in several in vitro and FEA models [13,28,36,38,41,43,44]. These findings indicate that FDP biomechanics depend on the support system as well as on the restorative material.

Material effects were more mixed. Zirconia often reported higher fracture resistance or lower modeled abutment stress than selected comparator materials, but this effect was not uniform across zirconia generations, connector configurations, retainer designs, and loading conditions [23,24,26,32,34]. Lithium disilicate, zirconia-reinforced lithium silicate, PICN, resin composite, and fiber-reinforced composite showed material-dependent behavior, but the evidence did not support one universal material ranking across all tooth-supported FDP designs [13,23,24,25,26,34].

Retainer design findings were also design-dependent. Full-coverage retainers showed higher pull-off retention in one tensile model, whereas conservative resin-bonded, inlay-retained, wing-retained, and cantilever designs were more sensitive to adhesive-interface behavior, connector geometry, and abutment selection [21,23,24,27,41,42]. The available evidence did not support one universal conservative-retainer hierarchy.

The most uncertain areas were clinical translation, reinforced tooth-supported zirconia concepts, titanium-bar-supported tooth-supported zirconia FDPs, and material-specific connector thresholds. These topics were either not directly tested in the included primary studies or were supported only by model-dependent laboratory or computational evidence.

## 4. Discussion

### 4.1. Main Interpretation of the Results

The evidence included indicates that tooth-supported FDP biomechanics are shaped by the combined effects of material, connector design, retainer form, span, loading, and abutment support [24,25,35,43,45]. This is the main contribution of the present review: it treats tooth-supported FDPs as material-design systems rather than as material groups alone [33,35,36]. In the in vitro evidence, mechanical risk often concentrated at the connector or retainer-connector transition [25,27,31,34]. In the FEA evidence, stress distribution changed with abutment tooth selection, alveolar support, connector dimensions, and loading direction [17,42,43,44]. The final included evidence base did not contain eligible clinical studies, so these findings should be read as laboratory and computational evidence rather than clinical proof.

### 4.2. Material Systems and Restorative Design

The material evidence should be interpreted by material class rather than as a single direct ranking. Zirconia-based systems formed the largest group, but they included different zirconia generations and design concepts [23,31,32,33]. Monolithic zirconia models were affected by connector area, connector contour, framework thickness, span, and zirconia type [23,33,45]. Veneered, semi-monolithic, cutback, or framework-modified zirconia concepts added veneer-support and interface variables, which means their behavior cannot be reduced to framework material alone.

Glass-ceramic and zirconia-reinforced lithium silicate comparators were represented in fewer designs. Their behavior depended on connector geometry, retainer configuration, and loading direction, and the included evidence did not support a general clinical ranking against zirconia. This supports a cautious material message: the same material may behave differently when the connector, retainer, pontic span, or support condition changes [17,26,35].

PICN, resin composite, and fiber-reinforced composite were represented mainly as comparator or conservative-design materials. These materials showed different stress-transfer patterns from zirconia and lithium disilicate, but the results were model-dependent and should not be interpreted as clinical thresholds [13,24,35].

Metal-based, graphene-based, and modified substructure comparators were too heterogeneous for a material-class conclusion [38,41,48]. Overall, the included evidence supports a context-specific material effect rather than a universal material ranking across tooth-supported FDP designs.

### 4.3. Conservative Retainer Designs

Conservative FDP designs shifted the biomechanical problem from bulk fracture alone toward connector integrity, adhesive retention, retainer extension, and local stress concentration [24,25,28,43]. Resin-bonded, inlay-retained, wing-retained, cantilever, fixed-fixed, and single-retainer concepts were represented across the included evidence, but they did not produce one consistent design hierarchy [24,25,28]. Retainer geometry affected fatigue behavior, debonding-related behavior, or stress distribution in several models, but the direction of effect depended on material, support, and loading setup [46,47]. Retainer fracture as a separate dominant outcome was not reported consistently; the repeated concerns were debonding, connector fracture, and stress concentration at the retainer-connector-abutment complex.

### 4.4. Connector, Framework, and Abutment Stress Distribution

Connector design was the most consistent biomechanical theme in the included evidence [17,31,34,35]. Larger connector dimensions often improved fracture resistance or reduced modeled connector stress, but connector area alone was not enough to explain performance [26,31,33,45]. Connector height, width, aspect ratio, embrasure radius, and transition shape changed stress concentration or failure behavior [27,32,34]. Framework thickness and pontic span also changed the effect of the same connector concept, so connector values should not be transferred between designs without matching span, support, and loading method [23,29,44].

The included evidence suggests several design-specific connector patterns, but it does not support one universal connector dimension for each material. For monolithic zirconia posterior FDPs, larger connector cross-sectional areas or greater connector height generally reported better mechanical behavior in the tested laboratory configurations. In a four-unit 5Y-TZP posterior FDP model, a 4 mm connector height with a 12.6 mm^2^ cross-sectional area reported higher fracture loads than a 2 mm connector height with a 6.3 mm^2^ area [23]. In a Y-TZP three-unit framework model, 9 mm^2^ connectors reported higher failure loads than 5 mm^2^ connectors under the reported test setup [33]. In monolithic zirconia three-unit FDPs, 12 mm^2^ connectors reported higher fracture-resistance values than 9 mm^2^ connectors, and a rounded gingival embrasure was favorable compared with a sharp embrasure at 9 mm^2^ [31]. In anterior cantilever resin-bonded FDPs, the larger 5 × 4 × 1 mm connector was associated with higher adjusted fracture strength than the 4 × 2 × 1 mm connector, although the independent material effect was not statistically significant after adjustment [27]. In long-span anterior 4Y-TZP FDPs, however, 9 and 12 mm^2^ connectors did not differ significantly, and pontic or cantilever spread appeared more influential than connector area alone [29]. These findings indicate that zirconia connector design should not be reduced to cross-sectional area alone.

For lithium disilicate, the anterior cantilever resin-bonded FDP study reported higher fracture-strength values with the larger connector than with the smaller connector, but the adjusted analysis did not support a significant independent material effect [27]. In studies where connector dimensions were fixed, or where connector aspect ratio was varied together with other design factors, favorable dimensions could not be generalized across lithium disilicate, zirconia-reinforced lithium silicate, or other ceramic comparators because material, span, connector shape, and loading method differed [26,33]. For fiber-reinforced composite, available studies did not provide enough connector-dimension comparisons to define a favorable threshold. For PICN and resin composite, FEA models with connectors ranging from 4 to 12 mm^2^ reported material-dependent stress patterns, but these findings were model-dependent and should not be interpreted as clinical connector thresholds [13,35]. Overall, connector height, width, area, transition radius, retainer design, span, material stiffness, and load direction should be reported together.

The FEA studies add that the abutment and supporting tissues are active parts of the design system, not passive supports [13,39,43,44]. Reduced alveolar support increased stress or strain in anterior resin-bonded models, and different abutment choices changed modeled risk-related outputs [42,44]. Distal abutment inclination, abutment substrate, periodontal ligament modeling, bone support, load direction, and load magnitude also affected stress distribution [37,39]. These outputs included von Mises stress, maximum principal stress, strain, displacement, and cement-related stress, which limits direct numerical comparison across studies.

### 4.5. Methodological Interpretation of the Evidence

The in vitro evidence was useful for identifying failure patterns, but it was highly method-dependent. Extracted teeth, artificial teeth, metal dies, different cementation protocols, different aging protocols, and different loading regimens were used across studies [22,27,28,32]. Thermocycling, fatigue loading, static fracture testing, retention testing, and failure mode reporting were not standardized, so fracture loads should be compared only when testing methods are sufficiently similar [23,24,28]. Waldecker et al. reported that abutment material and support resilience affected the estimated behavior of inlay-retained FDP test setups, which supports caution when comparing simplified laboratory models [37].

The FEA evidence was useful for testing design sensitivity, but it depended on model assumptions [17,36,43]. Geometry source, material properties, periodontal ligament modeling, bone modeling, mesh reporting, convergence testing, contact definitions, boundary conditions, loading conditions, validation, and sensitivity analysis were not reported with the same detail across studies [35,38,45,46,48]. The combined experimental and computational studies strengthened qualitative pattern reading when fracture origin or photoelastic stress fields matched modeled high-stress regions, but they did not convert FEA into clinical validation [13,34,35].

Several factors may explain the recurring methodological limitations identified in the included studies. In vitro FDP studies used different specimen sources, including extracted teeth, artificial teeth, resin dies, metal dies, and other simulated abutment models. These substrates do not reproduce periodontal support, elastic response, bonding conditions, or tooth variability in the same way. Specimen availability may also limit sample size, randomization, and group balance. Aging protocols, thermocycling, fatigue loading, antagonist geometry, loading angle, and endpoint definitions differed across studies. These differences limit direct comparison of fracture-load values. Blinding and outcome-assessor reporting were also incomplete in several studies, although failure-mode classification and specimen handling may influence interpretation.

The FEA evidence had a different source of uncertainty. FEA can isolate variables that are difficult to test experimentally, but the results depend on geometry source, segmentation, material properties, mesh density, periodontal ligament modeling, cement-layer modeling, contact definitions, boundary conditions, and load direction. Validation and sensitivity analysis were often absent or partial. This limits confidence in absolute stress values and supports interpretation based on stress patterns rather than universal numerical thresholds.

Future in vitro FDP studies should report connector height, connector width, cross-sectional area, embrasure radius, framework thickness, retainer geometry, cement space, surface treatment, aging, loading direction, antagonist geometry, failure origin, and failure-mode classification. They should include sample-size rationale, randomization where feasible, masked outcome assessment, and transparent reporting of excluded specimens. Future FEA studies should report model source, segmentation workflow, material properties, periodontal ligament and bone modeling, cement-layer assumptions, mesh convergence, contact definitions, constraints, loading magnitude and direction, validation strategy, and sensitivity analysis. Where possible, FEA models should be linked to experimental validation or shared model files.

### 4.6. Comparison with Prior Literature

Previous literature has mainly addressed clinical survival and complication outcomes for tooth-supported FDPs. A recent review reported pooled 5-year survival and complication estimates for metal-ceramic, veneered zirconia, monolithic zirconia, lithium-disilicate, and other all-ceramic tooth-supported multiple-unit FDPs [1]. The present review addresses a narrower and more biomechanical question: it examines how connector geometry, retainer design, framework form, loading, and abutment support affect mechanical behavior in tooth-supported FDP models.

The zirconia-focused reviews support the need to interpret zirconia FDPs as design-dependent restorations rather than as a single material category. Raigrodski et al. reported that clinical zirconia FDP studies differed in framework design, veneering method, follow-up time, and connector dimensions, which limited direct comparison between studies [6]. Stefanescu et al. also noted incomplete reporting of connector dimensions in zirconia FDP clinical studies [7]. The present review adds a mechanical explanation for these clinical themes by showing that connector area, connector shape, framework thickness, veneer support, and span can change fracture behavior or stress distribution in zirconia-based tooth-supported models.

Prior lithium disilicate reviews also point to the importance of prosthesis design. Pieger et al. reported more favorable survival evidence for lithium disilicate single crowns than for lithium disilicate FDPs [10]. The present evidence cannot test clinical survival, but it supports a design-based reading of these findings because lithium disilicate and related glass-ceramic comparators were sensitive to connector geometry, loading, and FDP configuration in included in vitro and FEA models.

The literature on conservative FDPs focused mainly on survival, debonding, and fracture. Chen et al. reported that inlay-retained FDPs had acceptable short- and medium-term survival but were affected by debonding, veneer fracture, dentine hypersensitivity, and secondary caries [3,12]. It was also reported that all-ceramic resin-bonded FDPs were mainly affected by debonding and framework fracture, and that cantilevered all-ceramic resin-bonded FDPs had more favorable pooled outcomes than two-retainer designs in their review [14]. The present review extends this clinical discussion by linking conservative retainer performance to retainer extension, connector dimensions, adhesive-interface stress, abutment choice, and periodontal support in preclinical models.

The added value of the present synthesis is that it connects themes of clinical complications from prior reviews to modifiable biomechanical variables. Previous clinical reviews could identify survival, chipping, debonding, framework fracture, and material-specific complication patterns, but they could not isolate the mechanical role of connector height, connector width, connector morphology, retainer geometry, framework thickness, abutment selection, or reduced alveolar support [2,11,49,50]. The included in vitro and FEA studies indicate that these variables can alter fracture origin, fatigue behavior, peak framework stress, cement stress, periodontal ligament strain, or abutment stress distribution [10,17,34]. This does not establish clinical superiority for any material or design, but it narrows the clinical questions that future studies should test.

### 4.7. Certainty of the Biomechanical Evidence

The certainty of evidence was assessed qualitatively because the review included only in vitro and finite element studies and did not include eligible clinical studies as primary evidence (Table 6). Conventional clinical certainty frameworks were not applied because the outcomes were laboratory fracture behavior, fatigue behavior, retention, and computational stress or strain endpoints rather than patient-level survival or complication outcomes. Certainty was judged by directness to the review question, consistency across studies, methodological appraisal, validation or experimental support, and comparability of methods.

Connector geometry and span-related findings had the strongest preclinical support because they were repeated across several in vitro and FEA studies and were often linked to fracture origin or stress concentration. However, certainty for clinical translation remains low because these findings were based on laboratory or computational models. Material ranking, retainer hierarchy, and abutment-stress thresholds had lower certainty because the results varied by model, material class, connector design, loading condition, and support assumption. Evidence for reinforced tooth-supported zirconia concepts or titanium-bar-supported tooth-supported zirconia FDPs was very low because direct primary evidence was not identified.

The strongest conclusions of this review concern repeated preclinical patterns, especially the role of connector geometry, span, and support conditions. These conclusions are supported by both in vitro and FEA evidence, although the certainty for clinical translation remains low. Conclusions based mainly on FEA evidence, such as the influence of abutment choice, periodontal support, bone level, and stress transfer to supporting tissues, are more tentative because several FEA studies had limited validation, incomplete convergence reporting, partial sensitivity analysis, or simplified boundary and contact assumptions. Therefore, FEA findings are interpreted as stress-distribution hypotheses and design-sensitivity evidence, not as clinical outcome evidence.

### 4.8. Future Directions

The included evidence may help clinicians and researchers identify mechanically sensitive regions in tooth-supported FDP designs, especially connectors, retainer-connector transitions, cement interfaces, and support-dependent stress zones. These findings should not be used to claim clinical survival, success, complication reduction, or material superiority because the final included evidence was limited to in vitro and FEA studies. Finite element studies have also evaluated zirconia implant concepts, including customized root-analog zirconia implants [51]. However, these implant-specific models represent a separate biomechanical context and should not be extrapolated to tooth-supported FDPs.

Future studies should test material and design variables under more comparable conditions, with clear reporting of connector height, width, area, morphology, framework thickness, retainer geometry, span, aging, loading, and failure origin. Future FEA studies should report validation, convergence testing, sensitivity analysis, contact and constraint definitions, periodontal ligament modeling, and bone modeling more completely. The present review did not identify direct primary evidence for tooth-supported zirconia FDPs supported by titanium bars or internal reinforcement structures. Titanium-bar support is mainly known from reinforced or full-arch prosthetic concepts, especially implant-supported designs, which were excluded from this review and should not be generalized to tooth-supported FDPs. For tooth-supported FDPs, the more directly supported research direction is refinement of monolithic zirconia designs, including connector height, connector width, connector morphology, framework thickness, span control, retainer geometry, and abutment support. Additively manufactured dental ceramics may also be considered in future fixed-prosthodontic research. However, current clinical-readiness evidence is concentrated on crowns, veneers, and partial-coverage restorations rather than multi-unit tooth-supported FDPs [52]. Direct testing of printed ceramic FDPs, including connector geometry, retainer configuration, span, aging, and loading, is required before clinical interpretation.

Internal reinforcement concepts may remain relevant as future laboratory questions when long spans, reduced connector dimensions, or unfavorable support conditions create high-stress regions. However, these concepts require direct tooth-supported in vitro testing and validated computational modeling before clinical interpretation is possible.

### 4.9. Strengths, Limitations, and Final Synthesis

The main strength of this review is its focused synthesis of tooth-supported FDP biomechanics across materials, connectors, retainers, frameworks, loading conditions, and abutment support. This focus helps separate material effects from design effects, which previous clinical reviews could not isolate because they primarily addressed survival and complication outcomes.

The first limitation is that no eligible clinical study was included in the final evidence base. Clinical studies were screened during eligibility assessment, but no clinical report met the final inclusion criteria for the review question focused on biomechanical design variables and stress-distribution outcomes. Therefore, the findings support biomechanical interpretation, not clinical claims about survival, success, complication reduction, or material superiority.

The second limitation is that the protocol was not prospectively registered. Although the protocol was prepared before final synthesis and was deposited in the Open Science Framework during revision, the absence of prospective registration may reduce transparency and may increase the perceived risk that eligibility, extraction, or synthesis decisions were influenced by the available literature. To reduce this concern, the review reports the eligibility criteria, search syntax, screening flow, extraction fields, appraisal tools, and synthesis approach in detail. Nevertheless, lack of prospective registration remains a limitation.

The third limitation is the search strategy. The search focused on the last 10 years and used specific title, abstract, and keyword terms related to tooth-supported FDPs, materials, and biomechanical outcomes. This approach may have missed older foundational studies or studies that used different terminology for connector geometry, retainer design, fixed partial dentures, or stress analysis. The search window was selected to focus on contemporary CAD/CAM materials, current zirconia generations, recent conservative-retainer designs, and modern FEA reporting. The findings should therefore be interpreted as a synthesis of recent preclinical evidence rather than a complete historical review of all FDP biomechanics.

The fourth limitation is methodological heterogeneity. In vitro studies differed in specimen source, abutment simulation, cementation protocol, aging, thermocycling, fatigue loading, antagonist geometry, loading angle, and failure definition. FEA studies differed in geometry source, material properties, periodontal ligament modeling, bone modeling, mesh reporting, convergence testing, contact definitions, boundary conditions, loading conditions, validation, and sensitivity analysis. These differences prevented meta-analysis and limited direct numerical comparison across studies.

The fifth limitation is material heterogeneity. The included studies evaluated different zirconia generations and comparator materials, including lithium disilicate, zirconia-reinforced lithium silicate, PICN, resin composite, fiber-reinforced composite, and other materials. Because these materials were not tested in the same designs and under the same conditions, the review does not support one universal material ranking.

Finally, the certainty of clinical translation is low. Laboratory and computational findings can identify mechanically sensitive regions and plausible design effects, but they cannot establish clinical survival, success, or complication risk. Future studies should use standardized aging and loading protocols, complete connector and retainer reporting, validated FEA models, convergence testing, sensitivity analysis, and clinical studies designed to test clearly defined biomechanical variables.

## 5. Conclusions

The included evidence was limited to in vitro and finite element studies. No eligible clinical study was included as primary evidence. The findings therefore support biomechanical interpretation, not clinical claims about survival, success, or long-term complication reduction.

Zirconia was the most frequently evaluated material system. Most zirconia evidence in this review involved monolithic zirconia or zirconia framework models rather than long-term clinical comparisons of veneered, cutback, and monolithic FDP designs. In monolithic zirconia models, fracture behavior and stress distribution were affected by connector dimensions, connector shape, framework thickness, span, loading direction, and abutment support. Veneered, cutback, or framework-modified zirconia concepts were represented mainly through computational or framework-design studies. These studies reported that veneer support and framework geometry can change stress location, but they do not establish clinical superiority for any veneering strategy.

Lithium disilicate, zirconia-reinforced lithium silicate, fiber-reinforced composite, PICN, resin composite, and metal-ceramic or polymer-based comparators were less consistently represented. Their behavior depended on connector geometry, retainer configuration, support conditions, and loading protocol. The available evidence does not support a single material ranking across all tooth-supported FDP designs.

Connector design was the most consistent biomechanical factor. Larger connector dimensions, greater connector height, and rounded or less acute transition forms often improved fracture resistance or reduced modeled stress in the tested configurations. However, connector area alone was not sufficient to explain performance. Connector height, width, aspect ratio, embrasure morphology, span, framework thickness, material, and loading direction should be reported together.

Conservative tooth-supported FDPs, including resin-bonded, inlay-retained, wing-retained, and cantilever designs, were especially sensitive to retainer geometry, adhesive-interface behavior, connector dimensions, and abutment selection. The evidence did not support one universal conservative-retainer hierarchy across all materials and designs.

Finite element studies indicated that abutment choice, alveolar bone level, periodontal support, abutment inclination, substrate condition, and loading direction can change stress transfer to the restoration, abutment teeth, periodontal ligament, and supporting bone. These findings are model-dependent and require experimental or clinical validation.

Future studies should use standardized aging and loading protocols, complete reporting of connector and retainer geometry, explicit failure-mode classification, sample-size justification, validated finite element models, convergence testing, sensitivity analysis, and direct testing of reinforced tooth-supported FDP concepts when such designs are proposed.

## Figures and Tables

**Table 1:** The PICO framework is used to define the review question and eligibility process.

PICO Element	Operational Definition for This Review
Population or model	Tooth-supported fixed dental prosthesis models, including extracted-tooth laboratory models, artificial-tooth laboratory models, and computational models. Eligible models could include abutment teeth, periodontal ligament, alveolar bone, restorative structures, or combinations of these components.
Intervention or exposure	Material selection, material combination, connector design, retainer design, framework design, pontic span, prosthesis configuration, abutment configuration, and loading condition in tooth-supported fixed dental prostheses.
Comparator	Alternative restorative materials, alternative framework or retainer configurations, different connector dimensions or geometries, different prosthesis designs, different abutment-support conditions, or different loading conditions. Studies without a direct comparator were eligible when they provided usable biomechanical data for an eligible tooth-supported fixed dental prosthesis design.
Outcomes	Fracture load, fracture resistance, fatigue behavior, failure mode, chipping, connector fracture, framework fracture, retainer fracture, debonding, retention force, stress distribution, strain distribution, von Mises stress, maximum principal stress, displacement, and stress in abutment teeth, periodontal ligament, or bone.
Study designs considered	In vitro mechanical, fatigue, fracture resistance, and finite element analysis studies. Clinical studies were screened using the initial eligibility framework but were not considered primary evidence unless they evaluated an eligible tooth-supported fixed dental prosthesis design and a relevant biomechanical design variable.
Review question derived from PICO	In tooth-supported fixed dental prostheses, how do material selection, connector design, retainer design, and prosthesis configuration influence mechanical performance and stress transmission to abutment teeth and supporting tissues?

**Table 2:** Inclusion and exclusion criteria

Domain	Inclusion Criteria	Exclusion Criteria
Population or model	Extracted-tooth laboratory models, artificial-tooth laboratory models, and computational models of tooth-supported fixed dental prostheses. Models could include abutment teeth, periodontal ligament, alveolar bone, restorative structures, or combinations of these components.	Implant-supported prostheses, tooth-implant-supported prostheses, removable prostheses, purely implant-abutment studies, single crowns unless analyzed as fixed dental prosthesis retainers, and material coupon studies without a fixed dental prosthesis design.
Prosthesis type	Conventional full-coverage tooth-supported fixed dental prostheses, fixed partial dentures, resin-bonded fixed dental prostheses, inlay-retained fixed dental prostheses, wing-retained fixed dental prostheses, cantilever tooth-supported fixed dental prostheses, and anterior or posterior tooth-supported bridge designs.	Implant-supported full-arch prostheses, implant bars, implant-retained hybrid prostheses, removable partial dentures, overdentures, and orthodontic appliances.
Materials	Zirconia, monolithic zirconia, veneered zirconia, partially veneered zirconia, bilayer ceramic systems, lithium disilicate, fiber-reinforced composite, resin composite frameworks, metal-ceramic comparators, and high-performance polymers when used in a tooth-supported fixed dental prosthesis design.	Materials used only in isolated coupon testing without a fixed dental prosthesis design. Materials used only in implant-supported or removable prosthetic designs.
Material focus	Focused on zirconia while considering comparators. Non-zirconia materials were eligible when they directly informed the review questions.	Materials with no relevance to tooth-supported fixed dental prosthesis biomechanics.
Outcomes	Fracture load, fracture resistance, fatigue behavior, failure mode, chipping, connector fracture, framework fracture, retainer fracture, debonding, retention force, stress distribution, strain distribution, von Mises stress, maximum principal stress, displacement, and stress in abutment teeth, periodontal ligament, or bone.	Studies without a relevant mechanical, fatigue, fracture, retention, or stress-distribution outcome.
Study design	In vitro mechanical, fatigue, fracture resistance, and finite element analysis studies.	Clinical studies that did not evaluate an eligible tooth-supported fixed dental prosthesis design or relevant biomechanical design variable, case reports, expert opinion papers, letters, editorials, conference abstracts without sufficient data, narrative reviews as primary evidence, and systematic reviews as primary evidence.
Language	English-language full texts. Non-English records could be listed as potentially relevant if identified, but were not extracted unless a reliable translation was available.	Non-English full texts without reliable translation.
Year	Published within the defined 10-year search window.	

**Table 3:** Database-specific search syntax.

Database	Search Field Syntax	Core Search String
PubMed/MEDLINE	Title/Abstract	((“fixed dental prosthesis”[Title/Abstract] OR “fixed partial denture”[Title/Abstract] OR bridge*[Title/Abstract] OR “resin-bonded fixed dental prosthesis”[Title/Abstract] OR “resin-bonded bridge”[Title/Abstract] OR “inlay-retained”[Title/Abstract] OR “wing-retained”[Title/Abstract] OR cantilever[Title/Abstract]) AND (“tooth-supported”[Title/Abstract] OR “abutment tooth”[Title/Abstract] OR “abutment teeth”[Title/Abstract] OR “natural teeth”[Title/Abstract]) AND (zirconia[Title/Abstract] OR ceramic[Title/Abstract] OR “lithium disilicate”[Title/Abstract] OR “fiber-reinforced composite”[Title/Abstract] OR FRC[Title/Abstract] OR monolithic[Title/Abstract] OR veneered[Title/Abstract]) AND (biomechanic*[Title/Abstract] OR “stress distribution”[Title/Abstract] OR “finite element”[Title/Abstract] OR fracture[Title/Abstract] OR “fracture resistance”[Title/Abstract] OR connector*[Title/Abstract] OR retainer*[Title/Abstract] OR framework[Title/Abstract]))
Scopus	TITLE-ABS-KEY	TITLE-ABS-KEY(“fixed dental prosthesis” OR “fixed partial denture” OR bridge* OR “resin-bonded fixed dental prosthesis” OR “resin-bonded bridge” OR “inlay-retained” OR “wing-retained” OR cantilever) AND TITLE-ABS-KEY(“tooth-supported” OR “abutment tooth” OR “abutment teeth” OR “natural teeth”) AND TITLE-ABS-KEY(zirconia OR ceramic OR “lithium disilicate” OR “fiber-reinforced composite” OR FRC OR monolithic OR veneered) AND TITLE-ABS-KEY(biomechanic* OR “stress distribution” OR “finite element” OR fracture OR “fracture resistance” OR connector* OR retainer* OR framework)
Web of Science Core Collection	Topic	TS = (“fixed dental prosthesis” OR “fixed partial denture” OR bridge* OR “resin-bonded fixed dental prosthesis” OR “resin-bonded bridge” OR “inlay-retained” OR “wing-retained” OR cantilever) AND TS=(“tooth-supported” OR “abutment tooth” OR “abutment teeth” OR “natural teeth”) AND TS=(zirconia OR ceramic OR “lithium disilicate” OR “fiber-reinforced composite” OR FRC OR monolithic OR veneered) AND TS=(biomechanic* OR “stress distribution” OR “finite element” OR fracture OR “fracture resistance” OR connector* OR retainer* OR framework)
Dentistry and Oral Sciences Source	Title or abstract	(TI “fixed dental prosthesis” OR AB “fixed dental prosthesis” OR TI “fixed partial denture” OR AB “fixed partial denture” OR TI bridge* OR AB bridge* OR TI “resin-bonded fixed dental prosthesis” OR AB “resin-bonded fixed dental prosthesis” OR TI “resin-bonded bridge” OR AB “resin-bonded bridge” OR TI “inlay-retained” OR AB “inlay-retained” OR TI “wing-retained” OR AB “wing-retained” OR TI cantilever OR AB cantilever) AND (TI “tooth-supported” OR AB “tooth-supported” OR TI “abutment tooth” OR AB “abutment tooth” OR TI “abutment teeth” OR AB “abutment teeth” OR TI “natural teeth” OR AB “natural teeth”) AND (TI zirconia OR AB zirconia OR TI ceramic OR AB ceramic OR TI “lithium disilicate” OR AB “lithium disilicate” OR TI “fiber-reinforced composite” OR AB “fiber-reinforced composite” OR TI FRC OR AB FRC OR TI monolithic OR AB monolithic OR TI veneered OR AB veneered) AND (TI biomechanic* OR AB biomechanic* OR TI “stress distribution” OR AB “stress distribution” OR TI “finite element” OR AB “finite element” OR TI fracture OR AB fracture OR TI “fracture resistance” OR AB “fracture resistance” OR TI connector* OR AB connector* OR TI retainer* OR AB retainer* OR TI framework OR AB framework)

**Table 4:** Supplementary conservative-design search syntax.

Database	Search Field Syntax	Supplementary Search String
PubMed/MEDLINE	Title/Abstract	zirconia[Title/Abstract] AND (“resin-bonded”[Title/Abstract] OR “inlay-retained”[Title/Abstract] OR “wing-retained”[Title/Abstract] OR cantilever[Title/Abstract]) AND (“fixed dental prosthesis”[Title/Abstract] OR “fixed partial denture”[Title/Abstract]) AND (“stress distribution”[Title/Abstract] OR “finite element”[Title/Abstract] OR survival[Title/Abstract] OR success[Title/Abstract] OR fracture[Title/Abstract])
Scopus	TITLE-ABS-KEY	TITLE-ABS-KEY(zirconia) AND TITLE-ABS-KEY(“resin-bonded” OR “inlay-retained” OR “wing-retained” OR cantilever) AND TITLE-ABS-KEY(“fixed dental prosthesis” OR “fixed partial denture”) AND TITLE-ABS-KEY(“stress distribution” OR “finite element” OR survival OR success OR fracture)
Web of Science Core Collection	Topic	TS=(zirconia) AND TS=(“resin-bonded” OR “inlay-retained” OR “wing-retained” OR cantilever) AND TS=(“fixed dental prosthesis” OR “fixed partial denture”) AND TS=(“stress distribution” OR “finite element” OR survival OR success OR fracture)
Dentistry and Oral Sciences Source	Title or abstract	(TI zirconia OR AB zirconia) AND (TI “resin-bonded” OR AB “resin-bonded” OR TI “inlay-retained” OR AB “inlay-retained” OR TI “wing-retained” OR AB “wing-retained” OR TI cantilever OR AB cantilever) AND (TI “fixed dental prosthesis” OR AB “fixed dental prosthesis” OR TI “fixed partial denture” OR AB “fixed partial denture”) AND (TI “stress distribution” OR AB “stress distribution” OR TI “finite element” OR AB “finite element” OR TI survival OR AB survival OR TI success OR AB success OR TI fracture OR AB fracture)

**Table 5:** Characteristics of included studies.

Study	Evidence Stratum	Study Design	Prosthesis Model	Materials and Design Variables	Testing or Modeling Protocol	Outcomes and Reported Findings
Almasi et al., 2019 [34]	Combined	In vitro flexure test plus FEA	Three-unit Y-TZP fixed partial denture framework based on a clinical case for replacement of a right lower molar. Four connector-design groups were evaluated.	Y-TZP frameworks. Connector shape and area were varied: circular and elliptical connectors with 5 mm^2^ or 9 mm^2^ cross-sectional area. Sample codes were ZC5, ZE5, ZC9, and ZE9.	Experimental loading used three-point bending to failure. FEA used a 500 N load on the pontic crest, with fixed geometry at the inferior surfaces of the molar and premolar abutments.	Both 5 mm^2^ connector designs failed below 500 N, whereas the 9 mm^2^ designs failed at approximately 3 times that load. Approximate failure loads were 0.455 kN for ZC5, 0.345 kN for ZE5, 1.229 kN for ZC9, and 1.450 kN for ZE9. FEA showed more even stress distribution with larger connector cross-sectional area. Stress concentration occurred where the connector joined the cap and where the restoration roof joined the side wall. Experimental fracture origins corresponded to high-stress regions predicted by FEA.
Chen et al., 2025 [35]	Combined	FEA with photoelastic validation	Maxillary posterior three-unit fixed partial denture replacing the first molar. Abutments were the maxillary second premolar and second molar.	3Y-TZP zirconia, lithium disilicate, PICN, and resin composite. Connector cross-sectional areas were 4, 6, 8, 10, and 12 mm^2^. FPD thickness was 1 mm cervical, 1.5 mm occlusal, and 1 mm middle third. Cement layer thickness was 0.03 mm.	Two 200 N loading modes were simulated: three-point loading on twelve 1 mm^2^ occlusal areas and pontic loading on three 1 mm^2^ areas on the pontic central groove.	At a constant connector cross-sectional area of 8 mm^2^, zirconia produced the lowest abutment tooth stress, 2.4177 MPa. Compared with zirconia, abutment stress increased by 2.37% for lithium disilicate, 7.67% for PICN, and 13.16% for resin composite. Increasing connector cross-sectional area from 4 to 12 mm^2^ reduced abutment stress by 1.65% in zirconia and 1.54% in PICN, but increased abutment stress by 115.63% in resin composite. At 12 mm^2^, average PDL stress was 1.2807 MPa for zirconia, 1.2796 MPa for lithium disilicate, 1.2778 MPa for PICN, and 1.2760 MPa for resin composite. Photoelastic results were reported as consistent with FEA. Stress concentration was reported mainly at connectors and occlusal loading areas. Photoelastic analysis showed a larger high-equivalent-stress area for resin composite than for zirconia at the same connector cross-sectional area, and lower equivalent stress as connector cross-sectional area increased for the same material.
Kasem et al., 2022 [24]	Combined	In vitro fatigue and fracture testing plus FEA	Posterior cantilever resin-bonded fixed dental prosthesis replacing a mandibular premolar. Reported mandibular molars served as abutments. Two retainer designs were tested: D1 inlay ring retainer and D2 lingual coverage retainer.	Monolithic high-translucency 3Y-TZP zirconia, Katana HT, and fiber-reinforced composite, TriLor. CAD/CAM restorations had a 3 × 3 mm connector, fixed 20 micrometer marginal gap, 60 micrometer internal cement gap, and standardized premolar pontic dimensions.	Aging included storage in distilled water for 24 h, 10,000 thermocycles between 5 and 55 degrees C, then 240,000 cycles at 50 N and 1.6 Hz. Fracture loading used a 5 mm spherical antagonist at 0.5 mm/min until fracture or visible plastic deformation. FEA loads were adjusted to the experimental group failure loads: 505 N for D1Z, 345 N for D1F, 548 N for D2Z, and 375.10 N for D2F.	All specimens survived artificial aging. Failure loads were D1Z 505.00 ± 61.50 N, D1F 345.00 ± 42.33 N, D2Z 548.00 ± 75.63 N, and D2F 375.10 ± 53.62 N. Material had a statistically significant effect on failure load, *p* = 0.001. Retainer design was not significant, *p* = 0.060. The material by design interaction was not significant, *p* = 0.734. FEA showed higher tooth-structure stress in D1 than D2. Dentin stresses were 15.80 MPa for D1Z, 11.00 MPa for D1F, 6.40 MPa for D2Z, and 1.07 MPa for D2F. Enamel stresses were 11.23, 11.00, 4.00, and 0.98 MPa. Luting resin stresses were 9.84, 20.66, 24.09, and 29.01 MPa. D2 showed more favorable failure patterns. Unfavorable failure occurred in 30% of D1Z and 20% of D1F, but 0% of D2Z and D2F. SEM indicated zirconia fractures often started at the occlusal surface, mostly near the connector region, while FRC failures were often at the tooth-restoration interface with fiber cohesive and adhesive failure.
Zhu et al., 2024 [13]	Combined	FEA with photoelastic validation	Maxillary posterior three-unit FPD replacing the first molar, with the second premolar and second molar as abutments. The distal abutment, second molar, was modeled with mesial inclination angles of 0, 6, 12, 18, 24, and 30 degrees.	Zirconia, lithium disilicate, PICN, and resin composite. The FPD thickness was 1.5 mm occlusal, 1 mm axial, and 1 mm shoulder. Cement layer thickness was 0.03 mm. The connector was reported as circular. Connector size reporting used inconsistent unit wording.	Two loading conditions were used: 200 N three-point loading on the FPD and 120 N pontic loading. Loading points were 1 mm^2^ circles distributed according to occlusal anatomy. FEA modeled loading directions at 0, 6, 12, 18, 24, and 30 degrees.	Stress was reported at the FPD connectors, enamel shoulder collar, periapical area, and root bifurcation. Under three-point loading, zirconia showed the largest average equivalent stress on the FPD, 2.81 MPa, followed by lithium disilicate, 2.63 MPa, PICN, 2.31 MPa, and resin composite, 2.07 MPa. For zirconia, average FPD stress increased from 2.81 MPa at 0 degrees to 2.93 MPa at 6 degrees, 3.13 MPa at 12 degrees, 3.28 MPa at 18 degrees, 3.58 MPa at 24 degrees, and 3.88 MPa at 30 degrees. Under pontic loading, resin composite at 30 degrees produced a sudden increase in alveolar bone stress, with maximum equivalent stress of 1754.10 MPa and average equivalent stress of 2.38 MPa. Photoelastic analysis reported similar qualitative patterns. Under pontic loading, stress was mainly concentrated near periapical and root furcation areas, and stress concentration increased as distal abutment inclination increased.
Ab Ghani et al., 2025 [27]	In vitro	In vitro fracture-strength study of anterior cantilever RBFDPs	Anterior cantilever resin-bonded fixed dental prosthesis replacing an incisor, with one reported incisal abutment.	Zirconia, IPS e.max ZirCAD Prime, and lithium disilicate, IPS e.max CAD. Connector dimensions: 5 × 4 × 1 mm and 4 × 2 × 1 mm. Retainer thickness 0.5 mm.	24 h saline storage at 37 degrees C, 5000 thermocycles between 5 degrees C and 55 degrees C. Load applied to the pontic palatal surface at 45 degrees and 0.5 mm/min.	Larger connector dimensions were associated with higher adjusted fracture strength, 224.71 N versus 120.48 N, *p* = 0.02. Zirconia values were numerically higher than lithium disilicate values, but material was not statistically significant after adjustment, *p* = 0.096. Reported subgroup values included zirconia 269 ± 27 N and lithium disilicate 180 ± 83 N for larger connectors, and zirconia 237 ± 52 N and lithium disilicate 116 ± 25 N for smaller connectors. Most specimens fractured at the connector with retainers remaining cemented. One zirconia specimen with the larger connector had tooth fracture. Two lithium disilicate specimens with the larger connector decemented.
Abd-Elghany and Mohsen, 2025 [22]	In vitro	In vitro tensile bond-strength study	Three-unit fixed partial denture replacing the mandibular second premolar; first premolar and first molar abutments.	BruxZir solid zirconia three-unit bridges. Retainer designs: full coverage on sound tooth, full coverage with fiber post and core, endocrown, and Sharonlay.	Specimens immersed in 4% acetic acid at 80 degrees C for 18 h. Tensile pull-off test at 1 mm/min with a metallic hook beneath the connector/embrasure.	Full coverage reported the highest tensile bond strength, 431.82 ± 18.90 N. Post-and-core full coverage was 212.93 ± 5.84 N, endocrown was 181.97 ± 12.06 N, and Sharonlay was 156.92 ± 10.75 N. Overall difference was significant, *p* < 0.001, with significant pairwise differences. Outcome was dislodgement/debonding under tensile loading. Detailed adhesive/cohesive failure classification was not reported.
Al-Dwairi et al., 2023 [28]	In vitro	In vitro fatigue and fracture study of cantilever inlay-retained FDPs	Cantilever inlay-retained fixed dental prosthesis in a maxillary premolar model.	Two multilayer zirconias, IPS e.max ZirCAD Prime and Zolid Gen-X. Three retainer designs: short wings, long palatal wing, and long palatal wing with occlusal extension. Minimum connector area 12 mm^2^.	24 h storage at 37 degrees C, 5000 thermocycles, then 1,200,000 cycles at 49 N and 1.7 Hz. Surviving specimens were loaded vertically on the pontic with a 5 mm ball at 0.5 mm/min.	Final fracture load did not differ significantly by design, material, or interaction. Dynamic fatigue failures differed by material, with ZirCAD Prime reporting more failures during cyclic loading than Zolid Gen-X, *p* = 0.009. The highest mean failure load was reported for D2GX, 1203.2 ± 1021.0 N, and the lowest for D2ZP, 344.0 ± 84.9 N, but the final load analysis was not significant. D1 had more debonding. D2 tended toward palatal wing fracture and debonding. D3 tended toward abutment tooth fracture. Connector fracture was not observed.
Coello et al., 2022 [29]	In vitro	In vitro fatigue study of long-span anterior zirconia FDPs	Canine-to-canine maxillary anterior FDP with four incisor pontics.	4Y-TZP zirconia, IPS e.max ZirCAD MT Multi. Connector sizes 9 and 12 mm^2^. Anterior cantilever or pontic spread values of 7, 10, and 13 mm.	Loaded at 135 degrees. Ramp to 165 N, then sinusoidal fatigue cycling from 50 to 280 N at 30 Hz for up to 5 million cycles.	Nine of 42 prostheses fractured before 5 million cycles. Connector size was not significant (*p* = 1.00). The six-group comparison was not significant (*p* = 0.2338). A comparison of 7 mm cantilever spread versus 10 mm and 13 mm combined was significant (*p* = 0.0407). Fractures occurred during fatigue cycling. The detailed fracture origin was not reported.
ElShamoty et al., 2024 [23]	In vitro	In vitro thermomechanical aging and fracture-resistance study	Posterior four-unit monolithic 5Y-TZP FDP supported by first premolar and second molar preparations.	Monolithic 5Y-TZP, Ceramill Zolid FX. Connector heights: 4 mm and 2 mm; cross-sectional areas: 12.6 and 6.3 mm^2^. Retainer occlusal thickness 1 or 2 mm.	One week water storage at 37 degrees C, 400,000 mechanical cycles at 50 N and 1.24 Hz, with 4000 thermocycles between 5 degrees C and 55 degrees C. Static load to failure on molar pontic at 0.5 mm/min.	No failures occurred during aging. Static fracture loads were CH4OT2 1563 ± 229 N, CH4OT1 1443 ± 291 N, CH2OT2 1067 ± 193 N, and CH2OT1 737 ± 111 N. Connector height was significant, *p* < 0.001. Retainer occlusal thickness was significant, *p* = 0.002. Interaction was not significant, *p* = 0.132. The 4 mm connector groups mainly fractured through the molar pontic. The 2 mm connector groups fractured through one or more connectors, and the 2 mm connector with 1 mm occlusal thickness fractured all connectors, resulting in loss of one or both pontics.
Hadzhigaev et al., 2023 [30]	In vitro	In vitro fracture-resistance study of distal-abutment preparation design	Three-unit mandibular zirconia FDP replacing the lower right second premolar, with premolar and molar abutments.	Full-contour monolithic ZrO2, DD Bio ZX2. Distal abutment design compared classical shoulder preparation with endocrown preparation and a 2 mm retention cavity. Distal connector 9 mm^2^ elliptical; mesial connector 9 mm^2^ circular.	No mechanical or thermal artificial aging. Vertical load to pontic with 5 mm sphere, 1 N preload, then 5 N/s until fracture.	Overall mean fracture resistance was 1099.66 ± 386.98 N. Endocrown preparation reported 1254.3 ± 358.37 N, and classical preparation 954.9 ± 381.54 N. Difference was not statistically significant, reported as *p* = 0.087 or *p* = 0.09 depending on text section. Nineteen of 20 fracture lines were located at the distal connector, with one at the mesial connector.
Hafezeqoran et al., 2020 [31]	In vitro	In vitro fracture-resistance study of connector size and shape	Three-unit mandibular monolithic zirconia FDP extending from the first premolar to the first molar.	Monolithic zirconia, Sirona inCoris TZI. Connector areas 9 and 12 mm^2^. Gingival embrasure radius compared round, 0.9 mm, and sharp, 0.25 mm.	Static three-point bending load at pontic center. Thermocycling and dynamic aging were not reported.	Reported fracture resistance values were 1327.4 ± 196.37 N for 9 mm^2^ round, 1054.4 N for 9 mm^2^ sharp, 1599.8 ± 167.09 N for 12 mm^2^ round, and 1440 ± 159.05 N for 12 mm^2^ sharp. Rounded versus sharp was significant at 9 mm^2^, *p* = 0.007, but not at 12 mm^2^, *p* = 0.075. A larger area was significant for both round and sharp connector designs. Detailed fracture-mode categories were not reported.
Kasem et al., 2023 [25]	In vitro	In vitro fatigue and fracture study of cantilever resin-bonded FDPs	Cantilever resin-bonded FDP replacing a mandibular premolar with a molar abutment.	Zirconia, Katana HT, and zirconia-reinforced lithium silicate (Vita Ambria). Five retainer designs: one wing, two wings, inlay ring, lingual coverage, and occlusal coverage. Connector area standardized at 16 mm^2^.	Thermal aging and 240,000 cycles of dynamic loading at 50 N and 1.6 Hz, followed by static compressive loading on the pontic at 0.5 mm/min.	Two debondings occurred during aging in the zirconia one-wing group. Reported zirconia failure-load means were OW 124 ± 18.83 N, TW 232.60 ± 15.60 N, IR 505 ± 61.51 N, LC 548 ± 75.64 N. OC 627 ± 153.42 N. Reported ZLS2 means were OW 130.40 ± 19.63 N, TW 151 ± 63.52 N, IR 219 ± 63.92 N, LC 177 ± 20.64 N, and OC 230 ± 40.37 N. Zirconia was higher than ZLS2 except for the one-wing design, where the material comparison was not significant. Connector-region fracture and debonding were common. Some catastrophic tooth fractures were reported in zirconia retainer designs. Exact group attribution was not reported consistently.
Kim et al., 2022 [26]	In vitro	In vitro fracture-strength study of the connector aspect ratio and material	Three-unit FDP replacing a mandibular first premolar, supported by canine and second premolar abutments.	Two lithium disilicate materials, IPS e.max CAD and Amber Mill, and two 5Y-PSZ zirconias, 3M Lava Esthetic and Katana UTML. Connector cross-sectional area fixed at 16 mm^2^, with W = H, W < H, and W > H designs.	24 h water storage, 10,000 thermocycles, 200,000 mechanical cycles at 50 N and 2 Hz, then static loading at 0.5 mm/min.	Material, connector design, and their interaction significantly affected fracture strength, with *p* < 0.001, *p* < 0.001, and *p* = 0.008. A wider-than-high connector generally reduced fracture strength, except in IPS e.max CAD. For example, Katana UTML decreased from 842 ± 140 MPa in W = H to 543 ± 111 MPa in W > H. Fracture location varied by material and connector design, and distal connector fracture was commonly reported. Detailed fracture-site coding was not reported.
Lotfy et al., 2024 [32]	In vitro	In vitro flexural-strength study of zirconia material and connector design	Three-unit posterior zirconia FDP supported by mandibular first premolar and first molar metal dies.	Gradient zirconia, IPS e.max ZirCAD 3Y/5Y, and translucent zirconia, BruxZir Shaded 16 PLUS 4Y. Round and sharp connector designs with a 3 × 3 mm connector size.	Static load applied perpendicularly to the middle of the pontic with a 3 mm steel bar at 0.5 mm/min until failure. Cyclic aging and thermocycling were not reported.	Flexural strength differed among groups (*p* < 0.0001). BruxZir sharp was 578.77 ± 32.90 MPa, BruxZir round was 709.10 ± 82.27 MPa, ZirCAD sharp was 744.07 ± 67.04 MPa, and ZirCAD round was 964.78 ± 50.99 MPa. Tukey’s test did not show a significant difference between BruxZir sharp and BruxZir round (*p* = 0.1151) or between BruxZir round and ZirCAD sharp (*p* = 0.8941). Visual examination of fracture patterns was performed. Exact fracture-mode categories were not reported.
Subsomboon and Urapepon, 2023 [33]	In vitro	In vitro fracture-load study of zirconia type and connector configuration	Mandibular posterior three-unit FDP, with prepared second premolar and second molar abutments.	Katana ML 3Y-TZP, Katana STML 4Y-TZP, and Katana UTML 5Y-TZP. Connector configurations 4 × 2.25 mm and 3 × 3 mm, both 9 mm^2^.	Static vertical load applied with a 5 mm steel ball at the pontic center and 1 mm/min until fracture. Dynamic functional loading and aquatic environmental aging were not used.	3Y-TZP reported higher fracture loads than 4Y-TZP and 5Y-TZP, *p* < 0.05. Values were 2740.6 ± 469.2 N and 2718.7 ± 339.0 N for 3Y-TZP, 1868.3 ± 281.6 N and 1663.6 ± 372.7 N for 4Y-TZP, and 1588.0 ± 255.0 N and 1559.1 ± 110.0 N for 5Y-TZP. Connector configuration was not significant (*p* = 0.44), and the interaction was not significant (*p* = 0.74). All zirconia bridges showed catastrophic bulk fracture. Fractures initiated from occlusal pontic surfaces and extended to the connector base. Fractures always occurred at the base of the connector.
Alruthea, 2020 [38]	FEA	FEA only	Mandibular posterior fixed-fixed bridges. Three-unit and four-unit designs were modeled.	Zirconia and graphene-based CAD/CAM bridge material compared. Material properties were reported for zirconia and graphene.	600 N vertical force. Three-unit bridge loaded at the central groove of the pontic. Four-unit bridge loaded on the marginal ridges of the pontics.	Graphene-based models showed higher stress, deflection, equivalent elastic strain, and deformation than zirconia models. Three-unit models showed higher values than corresponding four-unit models. Stress concentration was reported at the middle of the bridge and at connectors between pontics and abutments.
Bakitian et al., 2020 [36]	FEA	FEA only	Three-unit tooth-supported posterior FDP with first premolar and first molar abutments and a second-premolar pontic.	Translucent zirconia framework and veneering porcelain. Designs included monolithic zirconia, 0.3 mm and 0.5 mm semi-monolithic veneering, cap support, and wave support.	300 N load applied at 10 degrees oblique direction over six occlusal points.	Framework and veneer design affected stress distribution. Cap-support design showed the smallest maximum principal stress in veneering porcelain. Wave design showed the lowest maximum shear at the zirconia-veneer interface. Maximum stress shifted by design. Some models concentrated stress in veneer, while cap-support and monolithic models concentrated stress in the cervical zirconia framework.
Campaner et al., 2021 [39]	FEA	FEA only	Posterior three-unit zirconia FDP with first molar and first premolar abutments and a central pontic.	Zirconia fixed partial denture over different core or substrate conditions: sound dentin, resin composite core, and metal core. Cement layer reported as 100 µm.	300 N axial load applied at the center of the pontic or occlusal surface of the second premolar.	Metal cores produced the highest tensile stress peak in the FPD, reported as 116.4 MPa. Resin composite cores produced the highest cement-layer stresses in the molar and premolar abutments. Highest tensile stress was reported in connector regions.
Dimashkieh et al., 2024 [40]	FEA	FEA only	Three-unit and four-unit mandibular posterior fixed partial denture models. Traditional and sleeve designs were compared.	Zirconia, E-max, and Celtra Duo were compared. Traditional versus sleeve retainer design was the main design variable.	Vertical 300 N and oblique 150 N at 45 degrees. Four-unit loading distributed across pontic regions.	Oblique loading produced cortical bone stresses 12 to 15% higher than vertical loading. Four-unit sleeve designs reduced cortical bone stresses up to 20% compared with traditional designs. Sleeve design also reduced cement-layer stresses. Stress patterns were evaluated in cortical bone, spongy bone, mucosa, cement, roots, and prosthesis bodies.
Lakhe et al., 2025 [45]	FEA	FEA only	Mandibular posterior monolithic zirconia FDPs with 3-, 4-, and 5-unit span lengths.	Monolithic 3 mol% Y-TZP zirconia. Connector cross-sectional areas of 12, 15, 18, and 21 mm^2^ were compared across 9, 16, and 23 mm spans.	Vertical loads of 324 N and 1270 N applied to the central fossa of the pontic over 2 mm^2^.	Longer spans and smaller connectors increased von Mises stress. For the 9 mm span at 1270 N, reported stresses decreased from 736 MPa at 12 mm^2^ to 420 MPa at 21 mm^2^. In the 23 mm span, smaller connectors exceeded the stated zirconia tensile-strength threshold under maximal loading. Highest stress was reported in connector regions.
Asadi Paein Lamooki et al., 2026 [46]	FEA	FEA only	Mandibular three-unit FDP replacing the mandibular right first premolar. Canine full-coverage abutment and second premolar endocrown abutment.	Lithium disilicate and zirconia compared. Residual wall height of the endocrown abutment was 3 mm or 4.5 mm. Resin cement thickness reported as 0.12 mm.	Buccal loading at 135 degrees: 140 N on canine and 200 N on premolars. Centric occlusal loading: 200 N on premolars.	Greater residual wall height was reported to reduce stress, and connector regions had the highest stress. Several residual-wall-height comparisons were inconsistent. Highest stresses were reported mainly at connector regions.
Miura et al., 2017 [41]	FEA	FEA only	Three-unit cantilever FDP simulating a missing mandibular first molar. First and second premolars were the abutments.	Yttria partially stabilized zirconia and high noble gold alloy frameworks. Abutment materials were dentin and brass. Framework design variables included buccolingual width expansion and occlusal height expansion.	1 N vertical load applied to the cantilevered pontic at the distal fossa.	Basic design showed the highest maximum principal stress in frameworks. The combined width and height design had about half the maximum principal stress of the basic design and was interpreted by the authors as reducing stress concentration. Abutment stresses were reported at the mesial cervical first premolar and occlusal area of the second premolar.
Muthukumar et al., 2024 [47]	FEA	FEA only	Three-unit zirconia fixed partial dentures comparing endocrown-retained and post-and-core or fiber-post-retained designs.	Zirconia FDP, resin cement, enamel, dentin, cortical and cancellous bone, PDL, and fiber post materials. Material properties were reported.	A 150 N occlusal load was simulated.	The endocrown-retained design reported lower von Mises stress than the post-and-core design, 176.35 MPa versus 298.29 MPa, with lower shear stress at the cement interface and lower stress on abutment teeth. Stress endpoints included crown, tooth, and cement interface regions. Exact peak locations were not reported.
Oishi et al., 2025 [42]	FEA	FEA only	Maxillary anterior resin-bonded zirconia FDPs. Two-unit cantilever designs and three-unit two-retainer design were compared under different alveolar bone levels.	Y-TZP zirconia framework with adhesive cement. Retainer thickness reported as 0.5 mm. Designs included central incisor or canine single abutment and a two-retainer configuration.	100 N applied to a point 1 mm below the incisal edge of each abutment from a 45-degree incisal direction.	In models with bone loss of 4 mm or more, two-unit cantilever designs had lower cement shear stress than the three-unit two-retainer design. Framework maximum principal stress was smaller in two-unit designs across models. PDL and trabecular bone strains were greater in the single-abutment designs in reduced bone conditions. Stress and strain endpoints were evaluated in framework, adhesive cement, PDL, cortical bone, and trabecular bone.
Mohd Osman et al., 2023 [17]	FEA	FEA only	Anterior cantilever ceramic RBFDP with central incisor abutment and lateral incisor pontic.	Lithium disilicate and zirconia compared. Connector shapes were rectangular and trapezoidal, with different dimensions and volumes.	100 N, 150 N, and 200 N applied to the palatal surface at 45 degrees.	Higher loads increased maximum equivalent stress. Lithium disilicate connector stresses exceeded material strength at 150 N and 200 N in all tested shapes and dimensions. Zirconia rectangular connectors withstood all tested loads, while smaller trapezoidal zirconia connectors exceeded strength limits. Connector regions were the main stress sites.
Patel et al., 2024 [43]	FEA	FEA only	Maxillary anterior cantilever zirconia RBFDP replacing the left lateral incisor. Central incisor and canine abutments were compared.	Y-TZP zirconia. Design variables were abutment tooth, central incisor versus canine, and connector dimensions, 3 × 3 mm versus 3 × 4 mm.	200 N load applied at 45 degrees to the long axis of the pontic or lateral incisor.	Canine abutment designs had lower framework strain, PDL strain, cement shear stress, and debonding-risk area than central incisor designs. The 3 × 4 mm connector showed lower stress and strain than the 3 × 3 mm connector. Stress and strain endpoints were evaluated in framework, PDL, and cement.
Sukumoda et al., 2020 [44]	FEA	FEA only	Maxillary anterior zirconia RBFDP with upper central incisor and canine abutments under different alveolar bone levels.	Zirconia framework, adhesive cement, enamel, dentin, PDL, cortical bone, and cancellous bone.	200 N applied at 45 degrees from tooth axis to the center of the pontic and palatal side of retainer.	Maximum principal stress in the framework increased from 25.33 MPa at normal bone level to 29.35 MPa at the lowest bone level. Cement shear stress and PDL strain increased with reduced alveolar bone height. Cement stress concentrated at the connector side and cervical central-incisor region as bone level decreased.
Waldecker et al., 2019 [37]	FEA	FEA only	Zirconia-ceramic inlay-retained fixed partial dentures. The study validates in vitro test setups against a clinical reference situation.	Zirconia-ceramic inlay-retained FDPs. Variables included abutment tooth material, tooth mobility or resilience, restoration design, load direction, and cement-layer stiffness.	Loading directions and test conditions varied as part of validation analysis.	All tested variables affected calculated fracture resistance. Resin teeth underestimated fracture resistance by up to 57%. Resiliently supported metal abutments provided the closest approximation to clinical conditions, around −6% to +15% compared with the clinical reference. The focus was validation of test condition effects rather than one anatomical peak-stress site.
Yossef et al., 2018 [48]	FEA	FEA only	Posterior inlay-retained fixed dental prosthesis replacing a mandibular first molar.	Full zirconia one-piece inlay-retained FDP compared with a modified design using Co-Cr substructure, porcelain coating, and adhesive resin-coated wings.	400 N compressive load applied to the buccal cusp.	Both alternatives were reported to produce von Mises stress distributions within safe limits. The zirconia prosthesis showed lower stresses in the reported conclusion. Stress was evaluated in prosthesis components and supporting teeth. Exact peak locations were not reported.

**Table 6:** Qualitative certainty summary for main biomechanical findings. Certainty was judged for biomechanical interpretation and clinical translation separately because the included evidence was limited to in vitro and finite element studies.

Biomechanical Factor	Evidence Base	Consistency	Main Methodological Concerns	Certainty for Biomechanical Interpretation	Certainty for Clinical Translation
Connector dimensions and shape	In vitro and FEA	Moderate consistency	Heterogeneous loading, aging, connector geometry, and outcome definitions	Low to moderate	Low
Span length and cantilever extension	In vitro and FEA	Moderate consistency	Different spans, load directions, support conditions, and endpoint definitions	Low to moderate	Low
Material class	In vitro and FEA	Mixed	Different materials were tested in different designs and under different loading conditions	Low	Very low
Retainer design	In vitro and FEA	Mixed	Adhesive protocols, retainer geometries, and failure definitions varied	Low	Very low
Abutment support and periodontal condition	Mainly FEA	Moderate pattern consistency	Model-dependent periodontal ligament and bone assumptions	Low	Very low

## Data Availability

No new data were created or analyzed in this study. Data sharing is not applicable to this article.

## Supplementary Materials

The following supporting information can be downloaded at: https://www.mdpi.com/article/10.3390/ma19132844/s1, Supplementary File S1: PRISMA 2020 checklist.

## Abbreviations

The following abbreviations are used in this manuscript:

Abbreviation	Full term
3Y-TZP	3 mol% yttria-stabilized tetragonal zirconia polycrystal
4Y-TZP	4 mol% yttria-stabilized tetragonal zirconia polycrystal
5Y-PSZ	5 mol% yttria-stabilized partially stabilized zirconia
5Y-TZP	5 mol% yttria-stabilized tetragonal zirconia polycrystal
AB	Abstract
AI	Artificial intelligence
CAD	Computer-aided design
CAD/CAM	Computer-aided design and computer-aided manufacturing
Co-Cr	Cobalt-chromium
EBSCO	Elton B. Stephens Company
FDP	Fixed dental prosthesis
FEA	Finite element analysis
FPD	Fixed partial denture
FRC	Fiber-reinforced composite
Hz	Hertz
kN	Kilonewton
MEDLINE	Medical Literature Analysis and Retrieval System Online
MPa	Megapascal
N	Newton
PDL	Periodontal ligament
PICN	Polymer-infiltrated ceramic network
PICO	Population, Intervention, Comparator, Outcome
PRISMA	Preferred Reporting Items for Systematic Reviews and Meta-Analyses
QUIN	Quality Assessment Tool for In Vitro Studies
RBFDP	Resin-bonded fixed dental prosthesis
ROBFEAD	Risk-of-bias Framework for Dental Finite Element Analysis
SEM	Scanning electron microscopy
TI	Title
TITLE-ABS-KEY	Title, abstract, and keywords
TS	Topic search
Y-PSZ	Yttria partially stabilized zirconia
Y-TZP	Yttria-stabilized tetragonal zirconia polycrystal
ZLS	Zirconia-reinforced lithium silicate
ZrO_2_	Zirconium dioxide
µm	Micrometer
