# Supplementary material for: Biomechanics of Tooth-Supported Fixed Dental Prostheses: Material Systems, Connector Design, Retainer Design, and Abutment Stress Distribution—A Systematic Review of In Vitro and Finite Element Evidence
Source: Materials (Basel). 2026 Jul 3;19(13):2844. doi: 10.3390/ma19132844 (PMC13363059; doi:10.3390/ma19132844)
Supplement: Supplementary file 1 [file materials-19-02844-s001.zip › materials-4380716-supplementary.pdf]

PRISMA 2020 Checklist

Section and Topic	Item #	Checklist item	Location where item is reported
**TITLE**			
Title	1	Identify the report as a systematic review.	Title page. The article type identifies the manuscript as "Systematic Review," and the title identifies the article as a systematic review of in vitro and finite element evidence.
**ABSTRACT**			
Abstract	2	See the PRISMA 2020 for Abstracts checklist.	Abstract.
**INTRODUCTION**			
Rationale	3	Describe the rationale for the review in the context of existing knowledge.	Section 1, Introduction. The rationale is presented in the context of tooth-supported FDPs, material systems, connector design, retainer design, and abutment support.
Objectives	4	Provide an explicit statement of the objective(s) or question(s) the review addresses.	Section 1, final paragraph, objective statement; Section 2.2, PICO Framework, explicit review question.
**METHODS**			
Eligibility criteria	5	Specify the inclusion and exclusion criteria for the review and how studies were grouped for the syntheses.	Section 2.2, PICO Framework, Table 1; Section 2.3, Eligibility Criteria, Table 2; Section 2.9, Data Synthesis, study grouping by evidence type and synthesis theme.
Information sources	6	Specify all databases, registers, websites, organisations, reference lists and other sources searched or consulted to identify studies. Specify the date when each source was last searched or consulted.	Section 2.4, Information Sources; Section 2.3 and the Abstract report the search window; Section 2.4 lists databases and supplementary sources
Search strategy	7	Present the full search strategies for all databases, registers and websites, including any filters and limits used.	Section 2.5, Search Strategy; Table 3, database-specific search syntax; Table 4, supplementary search syntax.
Selection process	8	Specify the methods used to decide whether a study met the inclusion criteria of the review, including how many reviewers screened each record and each report retrieved, whether they worked independently, and if applicable, details of automation tools used in the process.	Section 2.6, Study Selection, reviewer process, independent title/abstract screening, full-text assessment, and disagreement resolution; Sections 2.4 and 2.10 describe the supplementary role of AI tools.
Data collection process	9	Specify the methods used to collect data from reports, including how many reviewers collected data from each report, whether they worked independently, any processes for obtaining or confirming data from study investigators, and if applicable, details of automation tools used in the process.	Section 2.7, Data Extraction, structured extraction fields and reviewer process; Section 2.10 describes AI and generative AI tool use and human verification.
Data items	10a	List and define all outcomes for which data were sought. Specify whether all results that were compatible with each outcome domain in each study were sought (e.g. for all measures, time points, analyses), and if not, the methods used to decide which results to collect.	Table 1, outcomes; Table 2, eligible outcomes; Section 2.7, Data Extraction, in vitro and FEA outcome fields.

Section and Topic	Item #	Checklist item	Location where item is reported
	10b	List and define all other variables for which data were sought (e.g. participant and intervention characteristics, funding sources). Describe any assumptions made about any missing or unclear information.	Section 2.7, Data Extraction, general, in vitro, and FEA variables; missing or unclear data were recorded as "not reported."
Study risk of bias assessment	11	Specify the methods used to assess risk of bias in the included studies, including details of the tool(s) used, how many reviewers assessed each study and whether they worked independently, and if applicable, details of automation tools used in the process.	Section 2.8, Methodological Appraisal, QUIN and ROBFEAD tools; Section 3.2 and Figure 2 present the results.
Effect measures	12	Specify for each outcome the effect measure(s) (e.g. risk ratio, mean difference) used in the synthesis or presentation of results.	Sections 2.7 and 2.9; Table 5. No pooled effect measure was used. Study-specific numerical outcomes, including fracture loads, stress or strain values, retention values, failure frequencies, and p-values where reported, were summarized descriptively.
Synthesis methods	13a	Describe the processes used to decide which studies were eligible for each synthesis (e.g. tabulating the study intervention characteristics and comparing against the planned groups for each synthesis (item #5)).	Sections 2.3 and 2.9; Table 2. Studies were grouped for synthesis by evidence type and biomechanical theme, with in vitro and FEA evidence synthesized separately.
	13b	Describe any methods required to prepare the data for presentation or synthesis, such as handling of missing summary statistics, or data conversions.	Sections 2.7 and 2.9. Missing data were recorded as "not reported." No quantitative pooling or data conversions were performed because of heterogeneity.
	13c	Describe any methods used to tabulate or visually display results of individual studies and syntheses.	Figure 1, PRISMA flow diagram; Figure 2, methodological appraisal traffic light plots; Table 5, study characteristics and individual findings; Table 6, qualitative certainty summary.
	13d	Describe any methods used to synthesize results and provide a rationale for the choice(s). If meta-analysis was performed, describe the model(s), method(s) to identify the presence and extent of statistical heterogeneity, and software package(s) used.	Section 2.9, Data Synthesis. Stratified narrative synthesis was used because prosthesis designs, materials, connector dimensions, abutment configurations, aging protocols, loading conditions, outcome measures, and FEA assumptions were heterogeneous. No meta-analysis was performed.
	13e	Describe any methods used to explore possible causes of heterogeneity among study results (e.g. subgroup analysis, meta-regression).	Section 2.9, Data Synthesis; Sections 3.4 to 3.9, thematic synthesis; Section 4.5, methodological interpretation. Heterogeneity was explored narratively by evidence type, design variable, material system, span/support condition, and modeling or testing assumptions. No subgroup analysis or meta-regression was performed.
	13f	Describe any sensitivity analyses conducted to assess robustness of the synthesized results.	Not applicable. No sensitivity analyses were conducted because no meta-analysis or quantitative pooling was

Section and Topic	Item #	Checklist item	Location where item is reported
			performed; Section 2.9 explains the rationale for narrative synthesis.
Reporting bias assessment	14	Describe any methods used to assess risk of bias due to missing results in a synthesis (arising from reporting biases).	Not formally assessed. No reporting-bias assessment due to missing results was performed because no quantitative synthesis was conducted; Section 2.9 explains that pooling was not planned because of heterogeneity.
Certainty assessment	15	Describe any methods used to assess certainty (or confidence) in the body of evidence for an outcome.	Section 4.7, Certainty of the Biomechanical Evidence; Table 6. Certainty was assessed qualitatively for biomechanical interpretation and clinical translation. GRADE was not applied because the final evidence base was limited to in vitro and finite element studies.
**RESULTS**			
Study selection	16a	Describe the results of the search and selection process, from the number of records identified in the search to the number of studies included in the review, ideally using a flow diagram.	Section 3.1, Search Results; Figure 1, PRISMA flow diagram.
	16b	Cite studies that might appear to meet the inclusion criteria, but which were excluded, and explain why they were excluded.	Section 3.1, Search Results; Figure 1. Full-text exclusion reasons are grouped by category. Individual excluded studies were not cited in the manuscript.
Study characteristics	17	Cite each included study and present its characteristics.	Table 5, Characteristics of included studies. Each included study is cited and its evidence stratum, design, prosthesis model, materials/design variables, protocol, outcomes, and reported findings are presented.
Risk of bias in studies	18	Present assessments of risk of bias for each included study.	Section 3.2, Methodological Appraisal Results; Figure 2, QUIN and ROBFEAD traffic light plots.
Results of individual studies	19	For all outcomes, present, for each study: (a) summary statistics for each group (where appropriate) and (b) an effect estimate and its precision (e.g. confidence/credible interval), ideally using structured tables or plots.	Table 5, individual study outcomes and reported findings; Sections 3.4 to 3.9, narrative results by synthesis theme.
Results of syntheses	20a	For each synthesis, briefly summarise the characteristics and risk of bias among contributing studies.	Section 3.2, appraisal profile; Section 3.3, Evidence Profile; Sections 3.4 to 3.9, synthesis themes; Table 6, qualitative certainty summary.
	20b	Present results of all statistical syntheses conducted. If meta-analysis was done, present for each the summary estimate and its precision (e.g. confidence/credible interval) and measures of statistical heterogeneity. If comparing groups, describe the direction of the effect.	No statistical synthesis or meta-analysis was performed. Study-specific descriptive results are presented in Table 5 and summarized narratively in Sections 3.4 to 3.9.
	20c	Present results of all investigations of possible causes of heterogeneity among study results.	Sections 3.4 to 3.9 and Section 4.5. Possible causes of heterogeneity were discussed narratively by material,

Section and Topic	Item #	Checklist item	Location where item is reported
			connector and framework design, retainer design, span, support condition, loading, aging, and FEA assumptions. No formal subgroup analysis or meta-regression was performed.
	20d	Present results of all sensitivity analyses conducted to assess the robustness of the synthesized results.	Not applicable. No sensitivity analyses were conducted because no quantitative pooling was performed; Section 2.9 explains the narrative synthesis approach.
Reporting biases	21	Present assessments of risk of bias due to missing results (arising from reporting biases) for each synthesis assessed.	Not formally assessed. No assessment of reporting bias due to missing results was performed because no quantitative synthesis was conducted; limitations related to heterogeneity and reporting are discussed in Sections 4.5 and 4.9.
Certainty of evidence	22	Present assessments of certainty (or confidence) in the body of evidence for each outcome assessed.	Section 4.7, Certainty of the Biomechanical Evidence; Table 6. A qualitative certainty summary is presented for the main biomechanical findings and for clinical translation.
**DISCUSSION**			
Discussion	23a	Provide a general interpretation of the results in the context of other evidence.	Sections 4.1 to 4.6, Discussion; Section 4.9, final synthesis.
	23b	Discuss any limitations of the evidence included in the review.	Sections 4.5, 4.7, and 4.9 discuss limitations of the in vitro and FEA evidence, methodological limitations, certainty, and clinical translation.
	23c	Discuss any limitations of the review processes used.	Section 4.9, Strengths, Limitations, and Final Synthesis, including non-registration, search-window limitations, methodological heterogeneity, material heterogeneity, and limits of clinical translation.
	23d	Discuss implications of the results for practice, policy, and future research.	Section 4.8, Future Directions; Section 5, Conclusions.
**OTHER INFORMATION**			
Registration and protocol	24a	Provide registration information for the review, including register name and registration number, or state that the review was not registered.	Section 2.1, Review Design, states that the protocol was prepared before final synthesis but was not prospectively registered; Section 4.9 discusses lack of prospective registration as a limitation.
	24b	Indicate where the review protocol can be accessed, or state that a protocol was not prepared.	Section 2.1, Review Design, provides the Open Science Framework non-prospective protocol deposit link and access date.

PRISMA

PRISMA 2020 Checklist

Section and Topic	Item #	Checklist item	Location where item is reported
	24c	Describe and explain any amendments to information provided at registration or in the protocol.	No registration amendments are applicable because the review was not prospectively registered. The final evidence scope and absence of eligible clinical studies are described in Sections 2.1, 2.3, 3.1, and 4.9.
Support	25	Describe sources of financial or non-financial support for the review, and the role of the funders or sponsors in the review.	Funding statement; Acknowledgments; Section 2.10, Use of Artificial Intelligence and Generative Artificial Intelligence Tools. The manuscript reports no external funding and author responsibility.
Competing interests	26	Declare any competing interests of review authors.	Conflicts of Interest statement.
Availability of data, code and other materials	27	Report which of the following are publicly available and where they can be found: template data collection forms; data extracted from included studies; data used for all analyses; analytic code; any other materials used in the review.	Supplementary Materials statement; Data Availability Statement. Study-level extracted and synthesized data are presented in the manuscript tables. The PRISMA checklist is provided as Supplementary File S1. No analytic code was generated because no quantitative synthesis was performed.

From: Page MJ, McKenzie JE, Bossuyt PM, Boutron I, Hoffmann TC, Mulrow CD, et al. The PRISMA 2020 statement: an updated guideline for reporting systematic reviews. BMJ 2021;372:n71. doi: 10.1136/bmj.n71. This work is licensed under CC BY 4.0. To view a copy of this license, visit https://creativecommons.org/licenses/by/4.0/
